# Observation of the magnonic Dicke superradiant phase transition

**DOI:** 10.1126/sciadv.adt1691

**Published:** 2025-04-04

**Authors:** Dasom Kim, Sohail Dasgupta, Xiaoxuan Ma, Joong-Mok Park, Hao-Tian Wei, Xinwei Li, Liang Luo, Jacques Doumani, Wanting Yang, Di Cheng, Richard H. J. Kim, Henry O. Everitt, Shojiro Kimura, Hiroyuki Nojiri, Jigang Wang, Shixun Cao, Motoaki Bamba, Kaden R. A. Hazzard, Junichiro Kono

**Affiliations:** ^1^ Applied Physics Graduate Program, Smalley–Curl Institute, Rice University, Houston, TX 77005, USA.; ^2^ Department of Electrical and Computer Engineering, Rice University, Houston, TX 77005, USA.; ^3^ Ames National Laboratory, Ames, IA 50011, USA.; ^4^ Department of Physics and Astronomy, Rice University, Houston, TX 77005, USA.; ^5^ Department of Physics, Materials Genome Institute, Institute for Quantum Science and Technology, Shanghai University, Shanghai 200444, China.; ^6^ Department of Physics, National University of Singapore, Singapore 117551, Singapore.; ^7^ Department of Physics and Astronomy, Iowa State University, Ames, IA 50011, USA.; ^8^ Smalley–Curl Institute, Rice University, Houston, TX 77005, USA.; ^9^ DEVCOM Army Research Laboratory-South, Houston, TX 77005, USA.; ^10^ Institute for Materials Research, Tohoku University, Sendai 980-8577, Japan.; ^11^ Department of Physics, Yokohama National University, Yokohama 240-8501, Japan.; ^12^ Institute for Multidisciplinary Sciences, Yokohama National University, 79-5 Tokiwadai, Hodogaya-ku, Yokohama 240-8501, Kanagawa, Japan.; ^13^ Department of Physics and Astronomy, University of California, Davis, CA 95616, USA.; ^14^ Department of Materials Science and NanoEngineering, Rice University, Houston, TX 77005, USA.

## Abstract

Two-level atoms ultrastrongly coupled with single-mode cavity photons are predicted to exhibit a quantum phase transition, entering a phase in which both the atomic polarization and the photonic field are finite even without external driving. However, this phenomenon, the superradiant phase transition (SRPT), is forbidden by a no-go theorem due to the existence of the diamagnetic term. Here, we present spectroscopic evidence for a magnonic SRPT in ErFeO_3_, where the role of the photonic mode (two-level atoms) in the photonic SRPT is played by an Fe^3+^ magnon mode (Er^3+^ spins). The absence of the diamagnetic term in the Fe^3+^-Er^3+^ exchange coupling ensures that the no-go theorem does not apply. Ultrabroadband terahertz and gigahertz magnetospectroscopy experiments revealed the signatures of the SRPT in thermal equilibrium, a kink and a softening, respectively, of two spin-magnon hybridized modes at the critical point. Systems near this phase are expected to harbor large-scale squeezing, which will potentially provide a route to next-generation quantum technologies.

## Teaser

Magnetospectroscopy experiments reveal signatures of the magnonic superradiant phase transition in ErFeO_3_ in thermal equilibrium.

## INTRODUCTION

An ensemble of two-level atoms can exhibit coherence through cooperative interaction with a single-mode quantized radiation field. Such cooperative optical processes have been extensively studied since the pioneering work of Dicke (*1*) in the context of superradiance and have recently attracted much-renewed interest in cavity quantum electrodynamics (QED) (*2*–*4*), condensed matter physics (*5*–*7*), and quantum information science (*8*). While superradiance phenomena occur in excited or driven systems, recent cavity QED studies of materials have focused on thermal equilibrium modified by cavity-enhanced vacuum electromagnetic fields.

A profound consequence of the Dicke model is a quantum phase transition, superradiant phase transition (SRPT) (*9*, *10*), where when the strength of the cooperative light-matter coupling exceeds a critical value, a static coherent electric or magnetic field and a finite atomic polarization appear spontaneously and simultaneously. We use the term “quantum phase transition” to refer to a zero-temperature phase transition induced by changing a part of the Hamiltonian that does not commute with the rest of the Hamiltonian. Even at a finite temperature, while thermal fluctuations lead to classical critical scaling, intrinsically quantum phenomena persist. A marked example is the quantum squeezing (*11*) obtained near the SRPT not only at zero temperature but also at finite temperature.

**Fig. 1.**
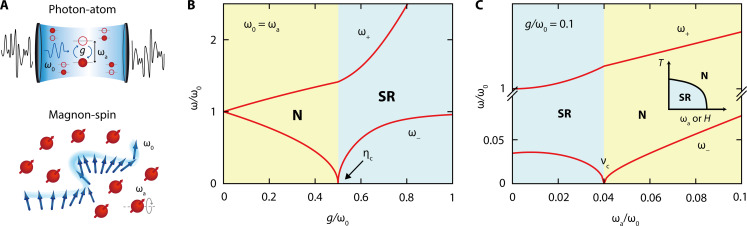
Comparison between a light-matter system and a magnon-spin system for the Dicke SRPT. (**A**) Top: A light-matter hybrid system with coupling strength *g* realized in a single-mode cavity with frequency ω_0_ containing an ensemble of two-level atoms with transition frequency ω_a_. Bottom: A magnon-spin hybrid system realized in ErFeO_3_. A single-mode magnon excitation of Fe^3+^ (an ensemble of Er^3+^ spins) plays the role of single-mode cavity photons (two-level atoms) in the Dicke model. (**B**) Normalized frequencies of the upper-polariton (ω_+_) and lower-polariton (ω_−_) modes as a function of *g*/ω_0_ calculated using the simple Dicke model at zero-detuning (ω_0_ = ω_a_) in the thermodynamic limit. When ω_−_ reaches zero, an SRPT occurs between the normal (N) and superradiant (SR) phases. (**C**) Normalized frequencies of ω_±_ modes as a function of ω_a_/ω_0_ calculated using the simple Dicke model with *g*/ω_0_ = 0.1 in the thermodynamic limit. When the lower-polariton frequency reaches zero, the system crosses the SR-N phase boundary. The inset illustrates the SR-N phase boundary when ω_a_/ω_0_ is tuned by an external DC magnetic field *H*.

Realization of the SRPT has been of much interest, but its occurrence in thermal equilibrium has been a subject of debate (*12*–*17*) due to the no-go theorem (*18*). Various methods have been proposed to circumvent the no-go theorem (*12*, *13*, *19*–*21*). At the core of the no-go theorem is the diamagnetic term (also known as the *A*^2^ term, where *A* is the electromagnetic vector potential) that inevitably appears in the minimal-coupling Hamiltonian describing the electric-dipole photon-atom interaction (Fig. 1A, top); this term adds positive energy to the system, causing the ground state to be robust against the SRPT (*15*, *18*, *22*, *23*).

A recent theoretical study has suggested that a magnonic version of the SRPT can occur in ErFeO_3_ via ultrastrong magnon-spin coupling because the nature of the coupling is spin-exchange interaction for which there is no *A*^2^ term (*21*).

Further, terahertz (THz) magnetospectroscopy experiments on a crystal of ErFeO_3_ have revealed ultrastrong coupling between a magnon mode of ordered Fe^3+^ spins and paramagnetic Er^3+^ spins (*24*). This system can be modeled by the Dicke model, where the Fe^3+^ magnon mode (the Er^3+^ spins) plays the role of the single cavity mode (the two-level atoms) of the Dicke model; see Fig. 1A (bottom). The Fe^3+^-Er^3+^ coupling exhibits Dicke cooperativity (*1*, *5*), i.e., the Fe^3+^-Er^3+^ coupling strength $\propto \sqrt{\rho }$, where ρ is the Er^3+^ spin density (*24*). More recently, a short-range atom-atom interaction (Er^3+^-Er^3+^ exchange interaction) has been incorporated for the simulation of an extended Dicke model (*25*). However, spectroscopic signatures of the SRPT, i.e., a polariton frequency softening down to zero and a concomitant change in the other polariton branch, have not been achieved to date.

Here, we report an experimental demonstration of the magnonic SRPT in ErFeO_3_ through magnetospectroscopy measurements in the THz and gigahertz (GHz) frequency ranges at low temperatures. We observed that, at the phase boundary between the superradiant (SR) phase and the normal (N) phase, the frequency of a branch of the Er^3+^ electron paramagnetic resonance (EPR) approaches zero while the frequency of an Fe^3+^ magnon displays a kink.

We developed an extended Dicke model, incorporating the single-ion anisotropy energy of Er^3+^ spins, which accurately reproduces the experimentally observed mode frequencies. The establishment of the magnonic SR phase will enable further experimental explorations of the nonintuitive vacuum-induced, squeezed ground states predicted for the SR phase (*26*–*28*) and of quantum information science (*29*) using magnonics (*30*, *31*). Our spectroscopic measurements are a key step in confirming the SRPT and understanding its nature before attempting entanglement measurements.

## RESULTS

### Expected spectroscopic signatures of the SRPT

The SRPT can be identified by the spectroscopic signatures of a simple Dicke model, describing a collection of *N* two-level atoms interacting with a single bosonic mode (*1*, *17*, *27*, *32*)

$${\hat{H}}^{\left(N\right)}/\hbar =\omega_{0}{\hat{a}}^{†}\hat{a}+\omega_{a}\left({\hat{S}}_{z}+\frac{N}{2}\right)+\frac{2g}{\sqrt{N}}\left({\hat{a}}^{†}+\hat{a}\right){\hat{S}}_{x}$$ (1)

where ${\hat{a}}^{†}$ ($\hat{a}$) is a bosonic creation (annihilation) operator of frequency ω_0_, ω_a_ is the transition frequency of the two-level atoms, ${\hat{S}}_{i}$ is a collective spin operator in the *i* direction, *N* is the number of two-level atoms, and *g* is the coupling strength between the bosonic mode and two-level atom. Later, we will derive a model similar to this from a spin Hamiltonian for a magnon-spin system containing extra terms that do not qualitatively change its eigenfrequencies and phase diagram. For a photon-atom system, one should also include proper diamagnetic or self-polarization terms to preserve the gauge invariance (*15*) and to recover the classical-quantum correspondence (*23*).

In the thermodynamic limit (*N* → ∞), through the Holstein-Primakoff transformation (*27*, *32*), Eq. 1 becomes $\hat{H}/\hbar =\omega_{0}{\hat{a}}^{†}\hat{a}+\omega_{a}{\hat{b}}^{†}\hat{b}+g\left({\hat{a}}^{†}+\hat{a}\right)\left({\hat{b}}^{†}+\hat{b}\right)$, where ${\hat{b}}^{†}$ ($\hat{b}$) is a bosonic creation (annihilation) operator corresponding to the atomic excitations ω_a_. Because this is a quadratic Hamiltonian, it can be analytically solved by a Bogoliubov transformation. Figure 1B plots the frequencies of the upper and lower polaritons, ω_+_ and ω_−_, respectively, normalized by ω_0_ as a function of *g*/ω_0_ at zero detuning, ω_0_ = ω_a_. The SRPT occurs, resulting in a complete frequency softening (a kink) of ω_−_ (ω_+_) at the phase boundary. In this case, we can also derive a condition for the SRPT at zero temperature

$$g>\frac{\sqrt{\omega_{a}\omega_{0}}}{2}$$ (2)

In the case of zero detuning (ω_a_ = ω_0_), this condition reduces to *g* > ω_0_/2, i.e., η_c_ = 0.5 is the critical value for the normalized coupling strength η ≡ *g*/ω_0_. One can imagine that the effect of *g* is largest when the light and atom degrees of freedom are on-resonant at zero detuning. Therefore, the standard strategy for realizing the SRPT is to maximize *g* to reach η_c_ = 0.5 for a fixed ω_0_ while maintaining zero detuning (ω_a_ = ω_0_); see Fig. 1B. However, even when η < 0.5, the SRPT can occur if one can reduce ω_a_ to satisfy Eq. 2 for fixed *g* and ω_0_. For example, when η = 0.1 (Fig. 1C), Eq. 2 becomes $\nu \equiv \omega_{a}/\omega_{0}<0.04$; that is, the SRPT occurs as a function of ν when it is decreased to the critical value ν_c_ = 0.04. In general, when η < 0.5 (η > 0.5), the SRPT occurs at ν_c_ < 1 (ν_c_ > 1), i.e., on the left (right) side of the zero-detuning point when ω_a_ is varied for fixed *g* and ω_0_; see section S1 and fig. S1 for details.

This nonstandard strategy aptly works for realizing a magnonic SRPT in ErFeO_3_. The Fe^3+^-Er^3+^ coupling strength *g* and the Fe^3+^ magnon frequency ω_0_ are nearly independent of the applied magnetic field, *H*, and their ratio is η < 1, while the Er^3+^ EPR frequency ω_a_ strongly depends on the applied DC magnetic field, via the Zeeman effect. Therefore, applying a magnetic field can tune ν. With realistic values of *g* and ω_0_ for ErFeO_3_ (*21*, *25*, *33*), the SRPT is expected to occur at a critical magnetic field, *H*_c_, when the temperature, *T*, is sufficiently low (inset to Fig. 1C). Notably, the critical temperature, *T*_c_, is maximum when ω_a_ = 0, i.e., when *H* = 0. As *H* increases, *T*_c_ decreases, and, hence, the SR phase is transformed into the N phase at *H* = *H*_c_(*T*) when *H* is varied at a constant temperature; as *T* is decreased, *H*_c_ monotonically increases from zero to a maximum value at *T* = 0, which is a quantum critical point. Figure 1C shows the frequencies of the two polariton branches, ω_±_, normalized by ω_0_, as a function of ν calculated using the simple Dicke model in the thermodynamic limit (*N* → ∞) where we assumed η = 0.1, which is a typical value found in the ultrastrong coupling regime (*3*, *4*, *6*).

### Magnetic structure of ErFeO_3_

**Fig. 2.**
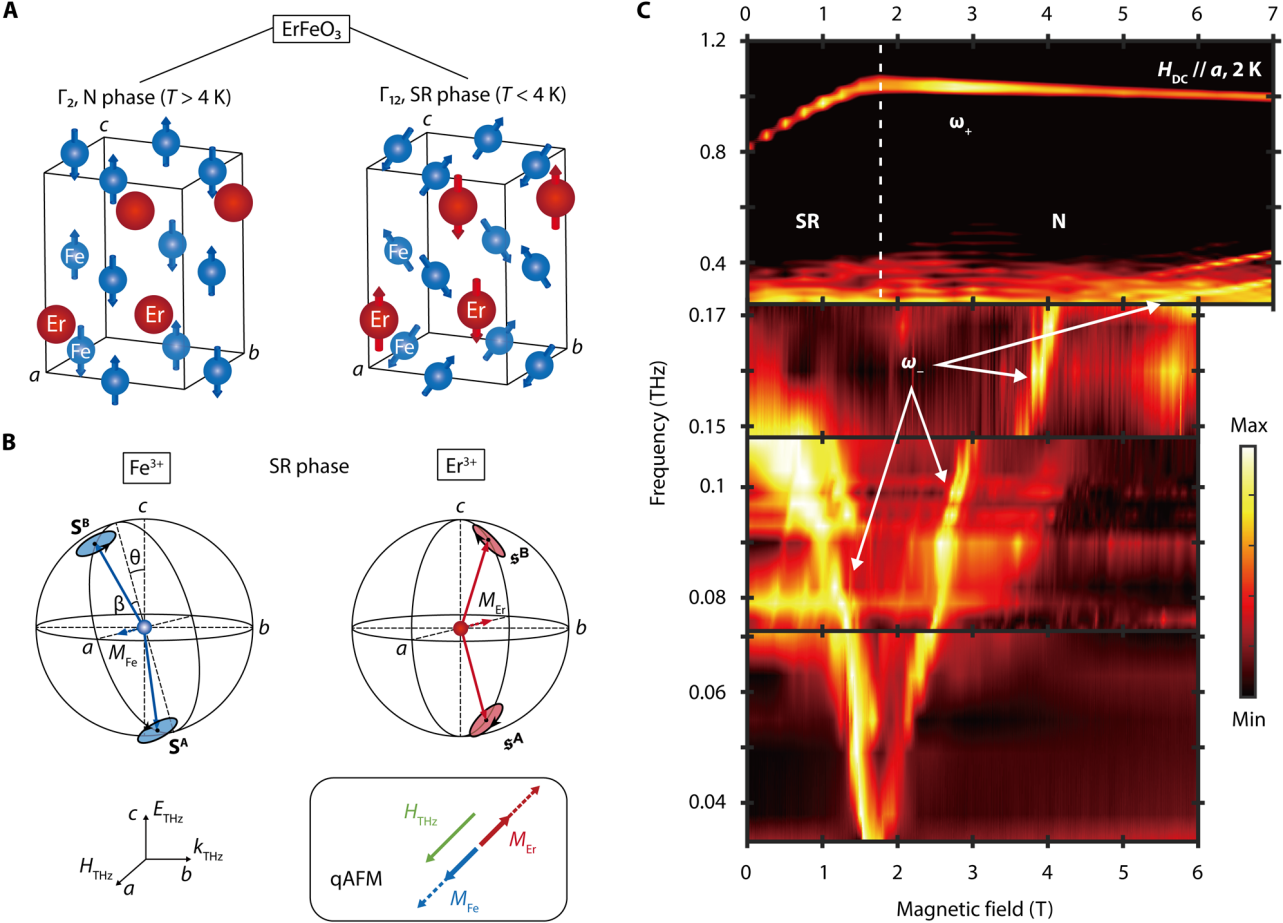
Spectroscopic evidence for the magnonic SRPT in ErFeO_3_. (**A**) Schematics of the magnetic structure of ErFeO_3_ in the Γ_2_ (N) phase and the Γ_12_ (SR) phase ($T<T_{N}^{\text{Er}}=4$ K). Er^3+^ spins become antiferromagnetically aligned at low temperatures, while the Néel vector of the Fe^3+^ spins rotates toward the *b* axis. (**B**) Spin dynamics of the qAFM of Fe^3+^ spins and Er^3+^ spins in the Γ_12_ phase triggered by a THz magnetic field polarized along the *a* axis. The magnitude of the net magnetization (*M*_Fe_ and *M*_Er_) oscillate (black box). The Fe^3+^ spins are ordered along the *c* axis and cant toward the *a* axis (β = 8.5 mrad). The plane where the Fe^3+^ spins lie is θ = 49° off from the *ac* plane. (**C**) Top: Transmission decrease ($1-\tilde{T}$) as a function of magnetic field in THz time-domain magnetospectroscopy (THz-TDMS), showing a kink in the upper polariton. Here, the maximum (minimum) value is 1 (0.7). Two middle: Temperature spectra in the sample upon continuous-wave (CW) GHz illumination with a base temperature of 2 K. The maximum temperature change is less than 90 mK. Here all spectra are scaled from 0 to 1. Bottom: Negative transmission spectra. Here, all spectra are scaled from 0 to 1. The GHz spectra in the bottom three panels show a softening in the lower-polariton frequency, which together with the kink in the upper-polariton mode, demonstrates the magnonic SRPT; see Fig. 1C.

When 4 K < *T* < 87 K, Fe^3+^ spins are antiferromagnetically ordered along the *c* axis with a canting toward the *a* axis by a small angle β (Γ_2_ in Bertaut’s notation) induced by the Dzyaloshinskii-Moriya (DM) interaction, which produces a weak ferromagnetic moment along the *a* axis (*34*). Figure 2A (left) shows the orthorhombic perovskite structure of ErFeO_3_ that consists of two Fe^3+^ and two Er^3+^ sublattices, described by the space group $D_{2h}^{16}-\text{Pbnm}$ with *a*, *b*, and *c* as the crystallographic axes that correspond to *x*, *y*, and *z* axes, respectively, in free space. As *T* decreases from 4 K, the Néel vector of Fe^3+^ spins continuously rotates toward the *b* axis (*35*), and paramagnetic Er^3+^ spins develop C-type antiferromagnetic order along the *c* axis (*36*) (Γ_12_ phase); see Fig. 2A (right). The definition of Γ phases is presented in table 1 of (*37*). A detailed description of its spin configuration in the Γ_12_ phase is presented in Fig. 2B. The two Fe^3+^ spins are denoted by ${\hat{S}}^{A}$ and ${\hat{S}}^{B}$, and the two Er^3+^ spins are denoted by ${\hat{s}}^{A}$ and ${\hat{s}}^{B}$. The plane where the Fe^3+^ spins exist has been rotated by θ = 49° with respect to the *ac* plane (*35*), while the Er^3+^ spins exist in the *ac* plane. *M*_Fe_ and *M*_Er_ are, respectively, defined as $S^{A}+S^{B}$ and $s^{A}+s^{B}$. Two order parameters for this phase transition can be defined as $\langle {\hat{S}}_{y}^{A/B}\rangle$ and $\langle {\hat{s}}_{z}^{A}-{\hat{s}}_{z}^{B}\rangle$, where ${\hat{S}}_{y}^{A/B}$ is the *y* component of the Fe^3+^ spins ${\hat{S}}^{A/B}$ and ${\hat{s}}_{z}^{A/B}$ is the *z* component of the Er^3+^ spins ${\hat{s}}^{A/B}$. The former captures the rotation of the Fe^3+^ spins toward the *b* (*y*) axis, while the latter captures the antiferromagnetic ordering of the Er^3+^ spins along the *c* (*z*) axis. This phase transition (Γ_2_ → Γ_12_) corresponds to the N → SR phase transition, i.e., the appearance of the static coherent electric or magnetic field and atomic polarizations in the context of a photonic SRPT (*21*). Below 4 K, the application of a magnetic field can induce a Γ_12_ → Γ_2_ transition (*38*, *39*), which we use in demonstrating the magnonic SRPT (SR → N).

### THz and GHz magnetospectroscopy studies of ErFeO_3_

We performed transmission magnetospectroscopy experiments on single crystals of ErFeO_3_ (see Materials and Methods for sample preparation) in the Voigt geometry in the THz and GHz photon frequency ranges to monitor the magnetic field evolution of the ω_±_ modes of this Fe^3+^-Er^3+^ hybrid system. The application of a static magnetic field, *H*_DC_, along the *a* axis continuously tuned the “bare” Er^3+^ EPR frequency ω_a_ via the Zeeman effect, whereas the bare Fe^3+^ magnon mode frequency ω_0_ was nearly independent of *H*_DC_. Here, the bare frequencies refer to the frequencies of the Fe^3+^ and Er^3+^ modes when uncoupled. The magnetic field moved the system out of the SR phase into the N phase at a critical field of μ_0_*H*_DC_ = 1.8 T at *T* = 2 K (where μ_0_ is the vacuum permeability).

In the THz frequency range (frequencies above 0.25 THz), we used THz time-domain magnetospectroscopy (THz-TDMS) (*40*) to monitor the ω_+_ mode; see Materials and Methods for experimental details. On the other hand, to monitor the ω_−_ mode in the GHz range, we used a set of continuous-wave (CW) devices (Virginia Diodes Inc.) producing single-frequency microwave radiation at frequencies below 172 GHz; see Materials and Methods for experimental details. From 33 to 71 GHz, we recorded the intensity of radiation transmitted through the sample as a function of magnetic field, which exhibited decreases at magnetic resonances. From 74 to 172 GHz, we monitored the sample temperature, which increased at resonance magnetic fields due to resonant absorption of microwave radiation; see fig. S8. Through these methods, we could locate the resonance frequencies of both the ω_+_ and ω_−_ modes as a function of magnetic field.

In the THz-TDMS experiments, the magnetic field component of the incident THz wave was set to be parallel to the *a* axis to access the quasi-antiferromagnetic (qAFM) magnon mode of Fe^3+^ while a static magnetic field was applied along the *a* axis (*33*); see the black box in Fig. 2B. The THz magnetic field parallel to the net magnetization direction triggered the out-of-phase spin precession in $S^{A/B}$ through the transient Zeeman torque, as shown in Fig. 2B. A *b*-cut sample made this configuration possible in the Voigt geometry. In this configuration, we found a pronounced absorption peak at 0.8 THz at 0 T, which we interpret as the ω_+_ mode.

The top panel of Fig. 2C shows the magnetic field dependence of this mode in transmission decreases ($1-\tilde{T}$), where $\tilde{T}$ is the transmittance. The data signal a clear phase transition at 1.8 T as a kink in the field evolution of its frequency. Below 1.8 T, frequency of the feature rapidly increased with *H*_DC_, whereas above 1.8 T, it slowly decreased with increasing *H*_DC_. This behavior is consistent with what is shown for the ω_+_ mode in Fig. 1C.

The bottom three panels of Fig. 2C display spectra taken in the GHz range, showing a marked softening behavior of the ω_−_ mode. At 0 T, a broad feature is seen at around 150 GHz, but it rapidly sharpens and red shifts as the applied magnetic field increases. The frequency of this mode eventually becomes lower than the lower bound of our frequency range but quickly reappears with increasing field, leaving a field gap of 0.2 T between the two peaks at 33 GHz. The middle of these peaks is located at 1.8 T, which agrees with the kink position of the ω_+_ mode in the top panel. This softening behavior is consistent with what is shown for the ω_−_ mode in Fig. 1C. A further increase of the field reveals a Zeeman-type response of the Er^3+^ spins, signifying the absence of antiferromagnetic order in the N phase. The ω_−_ mode eventually appears in THz absorption spectra in the top panel above 5.5 T, which confirms the consistency of our two different types of measurements. The less bright line that appears slightly above the ω_−_ mode does not play an important role in the SRPT (fig. S7).

### Mean-field theory of the spin Hamiltonian

Because no exact solution exists for a Dicke model that includes atom-atom (Er^3+^-Er^3+^ direct exchange) interaction, we model our experimental results with a spin Hamiltonian that has been widely studied for rare-earth orthoferrites (*21*, *41*, *42*), augmenting it with a term accounting for the anisotropy of the Er^3+^ spins. Then, we derived an extended Dicke model from the spin Hamiltonian to examine whether the *A*^2^ term appears and to elucidate the role of the Fe^3+^ magnon mode and Er^3+^ EPR in the context of light-matter interaction.

The system can be described by a spin Hamiltonian on a bipartite lattice with a unit cell consisting of two Fe^3+^ and two Er^3+^ spins (*21*, *24*). Nearest-neighboring spins interact via direct magnetic exchange. Owing to strong spin-orbit coupling in Fe^3+^, nearest neighbors of Fe^3+^ spins also have DM interactions between them. Both the Fe^3+^ and Er^3+^ spins are magnetically anisotropic, preferring the *xz* plane in our notation. Although the physical system has four Fe^3+^ spins and four Er^3+^ spins in a unit cell, the effective two-sublattice model can reproduce the resonance frequencies accurately because the Fe^3+^-anisotropic energies are much smaller than the DM interaction energy (*34*, *41*). The spin Hamiltonian can be written as

$${\hat{H}}_{\text{spin}}={\hat{H}}_{\text{Fe}}+{\hat{H}}_{\text{Er}}+{\hat{H}}_{\text{Fe}-\text{Er}}$$ (3)

where

$$\begin{array}{cc} {\hat{H}}_{\text{Fe}}= & \sum_{s=A,B}\sum_{i=1}^{N_{0}}\mu_{0}\mu_{B}g_{\text{Fe}}^{x}S_{i,x}^{s}H_{x}^{\text{DC}}+J_{\text{Fe}}\sum_{i,i\prime }S_{i}^{A}\cdot S_{i^{\prime }}^{B}\\ & -D_{\text{Fe}}^{y}\sum_{i,i\prime }\left(S_{i,z}^{A}S_{i^{\prime },x}^{B}-S_{i^{\prime },z}^{B}S_{i,x}^{A}\right)\\ & -\sum_{s=A,B}\sum_{i=1}^{N_{0}}\left[A_{\text{Fe}}^{x}{\left(S_{i,x}^{s}\right)}^{2}+A_{\text{Fe}}^{z}{\left(S_{i,z}^{s}\right)}^{2}\right], \end{array}$$ (4)

$$\begin{array}{cc} {\hat{H}}_{\text{Er}}= & \sum_{s=A,B}\sum_{i=1}^{N_{0}}\mu_{0}\mu_{B}g_{\text{Er}}^{x}s_{i,x}^{s}H_{x}^{\text{DC}}+J_{\text{Er}}\sum_{i,i\prime }s_{i}^{A}\cdot s_{i^{\prime }}^{B}\\ & -\sum_{s=A,B}\sum_{i=1}^{N_{0}}\left[A_{\text{Er}}^{x}{\left(s_{i,x}^{s}\right)}^{2}+A_{\text{Er}}^{z}{\left(s_{i,z}^{s}\right)}^{2}\right], \end{array}$$ (5)

and

$${\hat{H}}_{\text{Fe}-\text{Er}}=\sum_{i=1}^{N_{0}}\sum_{s,s\prime =A,B}\left[Js_{i}^{s}\cdot S_{i}^{s\prime }+D^{s,s\prime }\cdot \left(s_{i}^{s}\times S_{i}^{s\prime }\right)\right]$$ (6)

The $S_{i}^{A/B}$ and $s_{i}^{A/B}$ correspond to Fe^3+^ (*S* = 5/2) and Er^3+^ (s=1/2) spin operators at the *i*th site in the A/B sublattice; $\sum_{i,i\prime }$ represents a sum over the appropriate nearest neighbors; $J_{\text{Er}}$, $J_{\text{Fe}}$, and *J* are the direct exchange coupling strengths between Er^3+^-Er^3+^, Fe^3+^-Fe^3+^, and Fe^3+^-Er^3+^ spins, respectively; $D_{\text{Fe}}^{y}$ and $D^{s,s\prime }$ are the DM interaction strength between Fe^3+^-Fe^3+^ and Fe^3+^-Er^3+^ spins, respectively; $A_{\text{Fe}}^{x/z}$ and $A_{\text{Er}}^{x/z}$ are the anisotropy strengths of Fe^3+^ and Er^3+^ spins, respectively, along the *x*/*z* axes; $g_{\text{Fe}/\text{Er}}^{x}$ are the *x* component of the Landé *g*-factor tensors for Fe^3+^ and Er^3+^ spins, which are assumed to be diagonal; μ_B_ is the Bohr magneton. We included Er^3+^-anisotropy terms in Eq. 5, which were neglected in previous studies (*21*, *24*). Consistent with the inclusion of this term, magnetization measurements (*43*, *44*) suggest that Er^3+^ anisotropy is strong in this material. The inclusion of the terms is further necessitated by its large value compared to the exchange interaction between the Er^3+^ spins, i.e., $A_{\text{Er}}^{x/z}>J_{\text{Er}}$. Including the Er^3+^-anisotropy terms in the Hamiltonian produces a noticeably improved fit to the experimental data.

We note that, according to recent neutron scattering experiments performed on ErFeO_3_ below 4 K, the dispersion of the Er^3+^ spin excitation is found to be surprisingly weak even below 4 K (*36*).

**Fig. 3.**
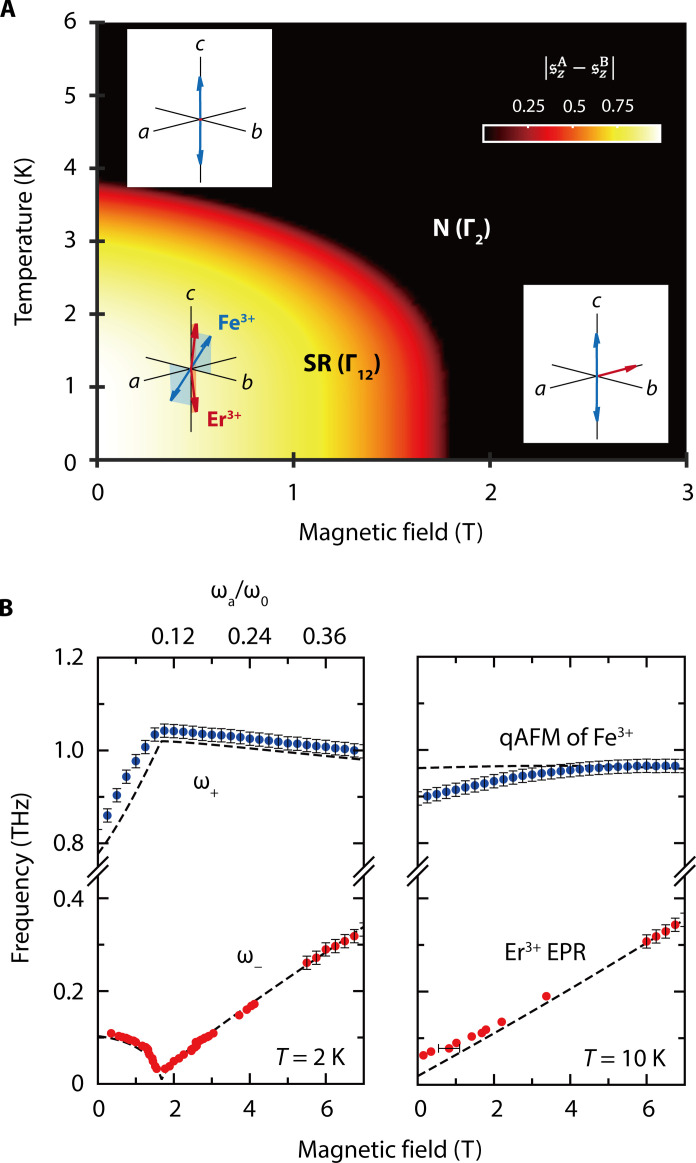
Mean-field calculation for the spin Hamiltonian of ErFeO_3_ in ${\vec{H}}_{\text{DC}}∥a$. (**A**) Theoretical *T*-*H* phase diagram. Insets: Schematics of the spin configuration in each phase. (**B**) Resonance frequencies of each spin subsystem as a function of the external magnetic field at 2 K (left) and 10 K (right). Dots, experimental results; dashed lines, calculated resonance frequencies. The top *x* axis on the left panel shows the SRPT occurs at the ratio of ω_a_/ω_0_ = 0.11. The vertical error bars indicate a frequency resolution determined by the time window (Δ*f* = 1/33 ps = 0.0286 THz). The horizontal error bar indicates an SD.

Figure 3A displays a mean-field *T*-*H* phase diagram of the spin Hamiltonian, for 0 ≤ *T* ≤ 6 K and 0 ≤ μ_0_*H* ≤ 3 T. This phase diagram, consistent with (*38*), shows the SR-phase boundary obtained by using $\langle {\hat{s}}_{z}^{A}-{\hat{s}}_{z}^{B}\rangle$ of Er^3+^ spins as the order parameter. One can also use $\langle {\hat{S}}_{y}^{A/B}\rangle$ of Fe^3+^ to construct the same phase boundary, because the two subsystems simultaneously order (fig. S11). The spin configurations are shown as an inset in Fig. 3A. The order parameters are obtained by performing a mean-field calculation on Eq. 3. The finite-temperature average of the spin operators then gives the self-consistency equations

$$\langle s_{∥}^{s}\rangle =-\frac{1}{2}\text{tanh}\left(\frac{g_{\text{Er}}\mu_{B}\left|{\bar{B}}_{\text{Er}}^{s}\right|}{2k_{B}T}\right)$$ (7)

$$\langle S_{∥}^{s}\rangle =-B_{S}\left(\frac{Sg_{\text{Fe}}\mu_{B}\left|{\bar{B}}_{\text{Fe}}^{s}\right|}{k_{B}T}\right)$$ (8)

where $B_{S}\left(x\right)=\frac{2S+1}{2S}\text{coth}\left(\frac{2S+1}{2S}x\right)-\frac{1}{2S}\text{coth}\left(\frac{x}{2S}\right)$ is the Brillouin function and $k_{B}$ the Boltzmann constant. BErs (BFes) are mean fields for the Er^3+^ (Fe^3+^) spins with s∈{A,B} with the assumption that spins are spatially uniform; see Eqs. 14 and 15.

Above 4 K at 0 T, the Fe^3+^ spins are antiferromagnetically ordered, while Er^3+^ spins are paramagnetic (Fig. 2A). As we cool down the system, the *b*-axis component of Fe^3+^ spins becomes finite through the rotation of the Néel vector around the *a* axis. This rotation behavior has been evidenced by nuclear magnetic resonance experiments (*35*). At the same time, Er^3+^ spins antiferromagnetically order along the *c* axis (Fig. 2A). When we apply an external magnetic field along the *a* axis stronger than *H*_c_, we break the antiferromagnetic ordering of Er^3+^ spins and restore the Fe^3+^ Néel vector along the *c* axis. These occur simultaneously due to the Fe^3+^-Er^3+^ antisymmetric exchange interaction that couples spins and magnons in the Dicke model as we discuss in the following.

Figure 3B shows the frequencies of both polariton branches, calculated on the basis of the spin Hamiltonian, below (2 K, left) and above (10 K, right) the critical temperature as a function of *H*_DC_, together with experimental results from Fig. 2C and fig. S6B. For the high-temperature measurements, see section S2. The calculated results are obtained by the linearized Heisenberg equations of motion

$$\hbar \frac{d}{\mathrm{dt}}{\delta s}^{s}=-{\delta s}^{s}\times g_{\text{Er}}\mu_{B}{\bar{B}}_{\text{Er}}^{s}\left(s^{A/B},S^{A/B}\right)-{\bar{s}}^{s}\times g_{\text{Er}}\mu_{B}B_{\text{Er}}^{s}\left({\delta s}^{A/B},\delta S^{A/B}\right)$$ (9)

$$\hbar \frac{d}{\mathrm{dt}}\delta S^{s}=-\delta S^{s}\times g_{\text{Fe}}\mu_{B}{\bar{B}}_{\text{Er}}^{s}\left(s^{A/B},S^{A/B}\right)-{\bar{S}}^{s}\times g_{\text{Fe}}\mu_{B}B_{\text{Er}}^{s}\left({\delta s}^{A/B},\delta S^{A/B}\right)$$ (10)

where ${\delta s}^{s}$ and $\delta S^{s}$ are the difference between the spin operators and their mean-field values. Then, resonance frequencies of Er^3+^ and Fe^3+^ spins are obtained by ${\delta s}^{s}=s^{s}\left(0\right)e^{i\omega_{\text{Er}}^{s}t}$ and $\delta S^{s}=\delta S^{s}\left(0\right)e^{i\omega_{\text{Fe}}^{s}t}$. A more detailed description is provided in Materials and Methods.

At 2 K, a good agreement was obtained by adjusting parameters in Eq. 3; see Materials and Methods, and table S1 for fitting. In particular, our calculations reproduced the observed ω_+_ kink and ω_−_ softening. At 10 K, we calculated the frequencies with the parameters obtained at 2 K. The two modes at 10 K do not display any critical behavior and are much less hybridized. Therefore, the two branches are essentially the qAFM mode of Fe^3+^ spins (∼ω_0_) and the EPR of Er^3+^ spins (∼ω_a_), respectively.

Note that the zero-field value of the Er^3+^ resonance is finite even at 10 K. This zero-field splitting is a result of the lifted degeneracy of the Er^3+^ ground state doublets (*24*, *45*) due to the symmetric Fe^3+^-Er^3+^ exchange interaction that exerts an internal effective magnetic field on Er^3+^ spins. Our fitting extracts the bare ω_0_ and ω_a_ appearing in the extended Dicke model, and, using these values, we indicate the value of ν = ω_a_/ω_0_ in the top *x* axis in the left panel, finding the critical ratio ν_c_ ∼ 0.11.

### Derivation of the extended Dicke model

We derived the extended Dicke model from the spin Hamiltonian, which manifests the role of the bare qAFM mode of Fe^3+^ (ω_0_) and bare Er^3+^ EPR (ω_a_) and the cooperative nature of coupling between the two modes, where no *A*^2^ term appears $\left[A\propto \left({\hat{a}}^{†}+\hat{a}\right)\right]$; see Materials and Methods for details. To derive the extended Dicke model, we first rewrite the Fe^3+^ subsystem ${\hat{H}}_{\text{Fe}}$ in terms of magnon annihilation and creation operators. Next, we apply the collective spin approximation on the Er^3+^ subsystem ${\hat{H}}_{\text{Er}}$. Last, we rewrite ${\hat{H}}_{\text{Fe}-\text{Er}}$ in terms of the spin-fluctuation operators of the Fe^3+^ spins and the collective spin operators of Er^3+^ spins. As a result, we end up having a Dicke-type Hamiltonian, which we call the extended Dicke model. In the model, we find the following three terms that compose the simple Dicke model

$$\frac{{\hat{H}}_{\text{Dicke}}}{\hbar }∼\omega_{0}{\hat{a}}^{†}\hat{a}+\omega_{a}{\hat{\Sigma }}_{x}^{+}+\frac{{\mathrm{ig}}_{z}}{\sqrt{N_{0}}}\left({\hat{a}}^{†}-\hat{a}\right){\hat{\Sigma }}_{z}^{-}+\ldots$$ (11)

where ${\hat{a}}^{†}$ ($\hat{a}$) is a qAFM magnon creation (annihilation) operator, $\omega_{a}=\mu_{0}\mu_{B}g_{\text{Er}}^{x}H_{x}^{\text{DC}}/\hbar$, ${\hat{\Sigma }}_{\xi }^{A/B}=\sum_{i=1}^{N_{0}}{\hat{s}}_{i,\xi }^{A/B}$ is a collective spin operator, ${\hat{\Sigma }}_{\xi }^{\pm }={\hat{\Sigma }}_{\xi }^{A}\pm {\hat{\Sigma }}_{\xi }^{B}$, and *N*_0_ is the number of unit cells. The Σ^−^ (Σ^+^) mode corresponds to the out-of-phase (in-phase) precession of Er^3+^ spins in the two sublattices. The additional terms in the extended Dicke model modifying the simple Dicke model are presented in Materials and Methods; see Eq. 39.

This model resembles the simple Dicke model in Eq. 1, which lacks the *A*^2^ term, and, therefore, the no-go theorem does not apply (*18*). $H_{x}^{\text{DC}}$ provides the tunability on the two-level transition energy through ω_a_. The third term captures the Dicke cooperative interaction through *N*_0_. We proved that *g*_*z*_ is the dominant contribution for the SRPT over other terms not shown here, in section S4. The form of *g*_*z*_ is provided in Eq. 43, and the origin of *g*_*z*_ essentially stems from the *x* component of the DM interaction between Fe^3+^ and Er^3+^ spins (*21*), i.e., the coupling between the two order parameters $\langle {\hat{S}}_{y}^{A/B}\rangle$ and $\langle {\hat{s}}_{z}^{A}-{\hat{s}}_{z}^{B}\rangle$. Together with the previous observation of the Dicke cooperativity (the coupling strength $\propto \sqrt{\rho }$) (*24*) and of hybridization of Fe^3+^ qAFM and the lower Er^3+^ EPR mode (see section S3), this direct correspondence concludes that our observation of the simultaneous kink and softening in ErFeO_3_ is the evidence of the SRPT. We note that the model is in the linear regime and vacuum regardless of the application of the magnetic field and is free from the gauge principle because the coupling stems from magnon-spin interactions, instead of photon-atom interactions where the gauge principle is important.

## DISCUSSION

We provided spectroscopic evidence that the Γ_12_ → Γ_2_ phase transition in ErFeO_3_ can be understood as the SR → N transition central to quantum optics (*21*). The simultaneous observation of the kink and softening of polariton branches at the same critical point is crucial for demonstrating the hybridization of the degrees of freedom and occurrence of the magnonic SRPT. This phase transition can also be regarded as the magnetic transition of the Jahn-Teller type (*46*, *47*). What makes our mode softening distinct from others found in various solids is the concomitant kink and applicability of the Dicke model. While our softening resembles a softening at a spin-flop transition (SFT), as found in MnF_2_ (easy-axis antiferromagnet) (*48*), the softening occurs without any concomitant kink because only one magnetic sublattice exists in the material. Furthermore, this softening occurs only when the external magnetic field is applied parallel to the Nèel vector (easy axis), in contrast to our work where the field is applied perpendicular to the Nèel vector (hard axis). Last, an SFT is typically a first-order phase transition that exhibits a discontinuous change, or a jump, in magnetization and may be accompanied by phase coexistence and hysteresis. On the other hand, the magnonic SRPT is a second-order phase transition where a continuous change occurs in magnetization. The Γ_12_ → #x0393;_2_ phase transition is a second-order phase transition where no discontinuous change is observed in magnetization measurements (*49*). Furthermore, an SFT occurs when a magnetic field (*H*_DC_) is applied parallel to the Néel vector. In this work, all measurements were performed with ${\vec{H}}_{\text{DC}}∥a$, meaning that *H*_DC_ was always applied perpendicular to the Néel vector of Er^3+^ spins (*c* axis), so an SFT was not a possibility. In conclusion, our softening does not stem from a simple SFT.

Our work is readily applied to other solids, such as other rare-earth orthoferrite or orthochromite compounds, where two different magnetic sublattices strongly interact with each other, which host different types of phase transitions (*50*–*52*). One can simulate different types of Dicke models or explore novel quantum vacuum phenomena at or in the SR phase by judiciously choosing candidate materials. We suggest two necessary conditions that must be satisfied for this analogy to be valid as a general guidance. First, one should find evidence that *g* exhibits the Dicke cooperative enhancement. Second, at the SR-phase boundary, one should find a simultaneous change in two magnetic sublattices that can be revealed by a kink and softening in spectroscopic measurements. Theoretical mapping of a spin Hamiltonian into Dicke models should be possible.

In conclusion, we observed the spectroscopic signatures of the magnonic SRPT in ErFeO_3_ in thermal equilibrium. Our work demonstrates the long-sought SRPT predicted by Hepp and Lieb in the Dicke model without the *A*^2^ term (*9*). The magnon-spin system opens up the possibilities to explore novel quantum vacuum phenomena predicted in the SR phase. At the SR-phase boundary of the Dicke model, a two-mode ground state becomes perfectly squeezed (*27*). This suggests that ErFeO_3_ in a magnetic field offers a unique opportunity to achieve large-scale quantum entanglement essential for quantum information science. Furthermore, our work provides insights into achieving photonic SRPTs through an exchange pathway with a readily achievable coupling strength in recent cavity QED, as well as a way to discover and control condensed matter phases based on powerful concepts developed in QED.

## MATERIALS AND METHODS

### Sample preparation

Polycrystalline ErFeO_3_ was first synthesized by a conventional solid-state reaction method using Er_2_O_3_ (99.9%) and Fe_2_O_3_ (99.98%) powders. According to the stoichiometric ratio, the original reagents were weighed carefully and pulverized with moderate anhydrous ethanol in an agate mortar. Mixtures were sintered at 1300°C for 1000 min and then cooled to room temperature. The sintered powders were thoroughly reground and pressed into a rod that was 70 mm in length and 6 mm in diameter by a Hydrostatic Press System (Riken Seiki Co. Ltd., model HP-M-SD-200) at 70 MPa and then sintered again at 1300°C for sufficient reaction. Single-crystal samples were then grown by an optical floating zone furnace (FZT-10000-H-VI-P-SH, Crystal Systems Corp; heat source: four 1-kW halogen lamps). Conditions like the melting power and the rate of sample rotation were stabilized and controlled in the molten zone.

### THz time-domain magnetospectroscopy

We performed THz-TDMS measurements in the Voigt geometry as we described in the “THz and GHz magnetospectroscopy studies of ErFeO_3_” section. A *b*-cut sample (1162 μm thick) was placed in a dry magneto-optical cryostat (Quantum Design, OptiCool) with variable temperatures *T* between 2 and 300 K and static magnetic fields μ_0_*H* up to 7 T. We generated THz pulses via optical rectification using a Ti:sapphire amplifier (795 nm, 40 fs, 1 kHz, Spectra-Physics, Spitfire Ace Pro XP) as a laser source that pumps a 1-mm-thick (110) zinc telluride (ZnTe) crystal, while detection was accomplished through electro-optical sampling in another ZnTe crystal with the same thickness.

### Thermal detection of EPR modes in static magnetic fields under GHz irradiation

We performed GHz magnetospectroscopy measurements in the Voigt geometry, as we described in the “THz and GHz magnetospectroscopy studies of ErFeO” section. Figure S8A shows the setup used for thermal detection of Er^3+^ EPR modes. We generated CW GHz waves using a diode (70 to 110 GHz, 25 mW, Virginia Diode, WR10SGX). For the frequency range of 140 to 220 GHz, a frequency multiplier (3.2 mW, WR5.1x2) was added. A 90 off-axis parabolic mirror collimated the GHz radiation beam that was incident onto the sample to ensure that the whole area of the sample (4 mm in diameter) was uniformly illuminated. A wire-grid polarizer defined the GHz magnetic field polarization direction to be along the *a* axis. A *c*-cut sample (800 μm thick) was placed in a dry magneto-optical cryostat (Quantum Design, OptiCool) at 2 K and static magnetic fields $\mu_{0}{\vec{H}}_{\text{DC}}∥a$ up to 6 T. A temperature sensor was located about 5 mm away from the sample, and they sat on the same copper plate. Temperature fluctuations of 1 mK or less were obtained with the temperature sensor (Lake Shore, CX-1050-CU-HT-1.4 M), and sensitivity of it is *dR*/*dT* = −1120.8 ohms K^−1^. We swept the magnetic field for each fixed frequency to 6 T with a step size of 0.02 T. At each magnetic field, we waited 5 s for the temperature to stabilize. We selected frequencies that showed a temperature change of 0 mK < Δ*T* < 90 mK at the peak temperature.

Figure S8B shows an example of thermal detection. With 90-GHz illumination, the sample temperature increased at some magnetic fields as we increased the magnetic field. This is because when the incident photon energy coincides with the transition energy of the spin resonances, nonradiative electron relaxation emitting phonons can happen, thereby increasing the lattice temperature. This technique has been used for observing electron cyclotron resonance in electron gases in semiconductors in the microwave regime (*53*). One of the advantages of using this method over the transmission measurement is that one can avoid the notorious standing wave effects that make the transmission curve more complicated.

Figure S9A shows experimental data at 2 K obtained through this method with frequencies ranging from 74 to 172 GHz, which are 0-to-1 scaled. These data correspond to those shown in the middle two panels in Fig. 2C. Above 95 GHz, side peaks appear at around 1.5 T. They are hybridized modes of the Er^3+^ EPR and a Fabry-Pérot cavity mode defined by the thickness of the crystal (*54*). For measurements at 10 K, this method does not work because the cryostat can maintain the temperature at 10 K even with GHz radiation. At 2 K, however, the cryostat is using its maximum cooling capability, and thereby any additional thermal load increases the temperature.

### GHz transmission spectroscopy experiments in pulsed magnetic fields

EPR measurements in pulsed magnetic fields with fixed frequencies ranging from 33 to 71 GHz and from 63 to 190 GHz were performed at temperatures of 2 and 10 K, respectively. Pulsed magnetic fields up to 6 T were applied parallel to the *a* axis. All curves have been 0-to-1 scaled.

At 2 K, we were able to observe the resonances as dips in transmission because the sample used for the pulsed magnetic field experiments was thinner (*d* = 200 μm) than the incident wavelengths (∼1 cm), allowing us to avoid the standing waves effect inside the sample (fig. S9A). Note that the dielectric constant of ErFeO_3_ is about 30. These data correspond to those shown in the bottom panel in Fig. 2C. Figure S9B shows transmission spectra at 10 K. These data are used in Fig. 3B (right). Here, because the thermal energy of 10 K (208 GHz) is higher than the transition energies, the population in the upper level is high, and, thereby, the transmission changes are minute. This results in high background noise at low frequencies after we do the 0-to-1 scale.

### Mean-field calculation of resonance frequencies and a phase diagram from the spin Hamiltonian

We calculated the ω_±_ by a combination of mean-field theory and linearization of the Heisenberg equations of motion of the spin operators, as in (*21*). Assuming that the average values of the spin operators, $S^{A/B}$ and $s^{A/B}$, are the same in all the unit cells, we self-consistently solved for the 12 mean fields, ${\bar{S}}^{A/B}$ and ${\bar{s}}^{A/B}$ in thermal equilibrium. The resonance frequency was obtained by linearizing the Heisenberg equations of motion of these 12 spin operators around the mean-field values and solving the resulting linear differential equations.

We calculate the resonance frequencies from the spin Hamiltonian with the method of (*21*). The Heisenberg equations of motion of the 12 spin operators are

$$\hbar \frac{ds^{s}}{\mathrm{dt}}=-s^{s}\times g_{\text{Er}}\mu_{B}B_{\text{Er}}^{s}\left(s^{A/B},S^{A/B}\right)$$ (12)

$$\hbar \frac{dS^{s}}{\mathrm{dt}}=-S^{s}\times g_{\text{Fe}}\mu_{B}B_{\text{Fe}}^{s}\left(s^{A/B},S^{A/B}\right)$$ (13)

dropping the unit cell index due to the assumption that spins are spatially uniform, with s∈{A,B}, and $B_{\text{Er}}^{s}$ ($B_{\text{Fe}}^{s}$) the mean fields for the Er^3+^ (Fe^3+^) spins

$$B_{\text{Er}}^{s}=B^{\text{DC}}+\frac{2z_{\text{Er}}J_{\text{Er}}}{\mu_{B}g_{\text{Er}}}s^{\bar{s}}+\frac{2}{\mu_{B}g_{\text{Er}}}\sum_{s\prime =A,B}\left[JS^{s}-\left(D^{s,s\prime }\times S^{s}\right)-A_{\text{Er}}\cdot s^{s}\right]$$ (14)

$$B_{\text{Fe}}^{s}=B^{\text{DC}}+\frac{2z_{\text{Fe}}J_{\text{Fe}}}{\mu_{B}g_{\text{Fe}}}S^{\bar{s}}-\frac{z_{\text{Fe}}}{\mu_{B}g_{\text{Fe}}}D_{\text{Fe}}\times S^{\bar{s}}+\frac{2}{\mu_{B}g_{\text{Fe}}}\sum_{s\prime =A,B}\left[JS^{s}-\left(D^{s,s\prime }\times S^{s}\right)-A_{\text{Fe}}\cdot s^{s}\right]$$ (15)

where $\bar{s}$ is the complementary sublattice of *s* and *z*_Fe_ = 6 is the number of nearest neighboring Fe^3+^ spins. $D^{s,s\prime }$ are expressed in terms of two values $D_{x}$ and $D_{y}$ as $D^{A,A}={\left(D_{x},D_{y},0\right)}^{t}$, $D^{A,B}={\left(-D_{x},-D_{y},0\right)}^{t}$, $D^{B,A}={\left(-D_{x},D_{y},0\right)}^{t}$, and $D^{B,B}={\left(D_{x},-D_{y},0\right)}^{t}$. Equation 15 is the same as that in (*21*), but Eq. 14 has an additional term coming from the inclusion of the Er^3+^ anisotropy.

The equations of motion are linearized around the equilibrium mean fields, 

${\bar{B}}_{\text{Er}/\text{Fe}}^{s}\equiv B_{\text{Er}/\text{Fe}}\left({\bar{s}}^{A/B},{\bar{S}}^{A/B}\right)$. These equations of motion correspond to the effective Hamiltonians for the Er^3+^ and Fe^3+^ spins

$$H_{\text{Er}}^{s}=g_{\text{Er}}\mu_{B}s^{s}\cdot {\bar{B}}_{\text{Er}}^{s}=g_{\text{Er}}\mu_{B}s_{∥}^{s}\left|{\bar{B}}_{\text{Er}}^{s}\right|$$ (16)

$$H_{\text{Fe}}^{s}=g_{\text{Fe}}\mu_{B}S^{s}\cdot {\bar{B}}_{\text{Fe}}^{s}=g_{\text{Fe}}\mu_{B}S_{∥}^{s}\left|{\bar{B}}_{\text{Fe}}^{s}\right|$$ (17)

where $s_{∥}$ ($S_{∥}$) is the spin operator in the direction parallel to the mean field.

The finite-temperature average of the spin operators then gives the self-consistency equations

$$\langle s_{∥}^{s}\rangle =-\frac{1}{2}\text{tanh}\left(\frac{g_{\text{Er}}\mu_{B}\left|{\bar{B}}_{\text{Er}}^{s}\right|}{2k_{B}T}\right)$$ (18)

$$\langle S_{∥}^{s}\rangle =-B_{S}\left(\frac{Sg_{\text{Fe}}\mu_{B}\left|{\bar{B}}_{\text{Fe}}^{s}\right|}{k_{B}T}\right)$$ (19)

where $B_{S}\left(x\right)=\frac{2S+1}{2S}\text{coth}\left(\frac{2S+1}{2S}x\right)-\frac{1}{2S}\text{coth}\left(\frac{x}{2S}\right)$ is the Brillouin function.

The linearized equations of motion are thus found to be

$$\hbar \frac{d}{\mathrm{dt}}{\delta s}^{s}=-{\delta s}^{s}\times g_{\text{Er}}\mu_{B}{\bar{B}}_{\text{Er}}^{s}\left(s^{A/B},S^{A/B}\right)-{\bar{s}}^{s}\times g_{\text{Er}}\mu_{B}B_{\text{Er}}^{s}\left({\delta s}^{A/B},\delta S^{A/B}\right)$$ (20)

$$\hbar \frac{d}{\mathrm{dt}}\delta S^{s}=-\delta S^{s}\times g_{\text{Fe}}\mu_{B}{\bar{B}}_{\text{Er}}^{s}\left(s^{A/B},S^{A/B}\right)-{\bar{S}}^{s}\times g_{\text{Fe}}\mu_{B}B_{\text{Er}}^{s}\left({\delta s}^{A/B},\delta S^{A/B}\right)$$ (21)

where ${\delta s}^{s}$ and $\delta S^{s}$ are the difference between the spin operators and their mean-field values.

These equations are solved to obtain the resonance frequencies. As coupled homogeneous linear differential equations with constant coefficients, they are solved by ${\delta s}^{s}=s^{s}\left(0\right)e^{i\omega_{\text{Er}}^{s}t}$ and $\delta S^{s}=\delta S^{s}\left(0\right)e^{i\omega_{\text{Fe}}^{s}t}$, where the frequencies ω and corresponding normal modes are found by solving a set of linear equations, iωs=Ms, where $s=\left(s^{A},s^{B},S^{A},S^{B}\right)$ and *M* is an anti-Hermitian matrix. The resonance frequencies are −i times the eigenvalues of *M*.

### Model fitting

The parameters in Eq. 5, namely, *J*_Er_, $g_{\text{Er}}^{x}$, $A_{\text{Er}}^{x}$, and $A_{\text{Er}}^{z}$, as well as $A_{\text{Fe}}^{z}$ and $g_{\text{Fe}}^{x}$ in Eq. 4, are used as fitting parameters to fit *T*_c_ and the spectroscopic data at *T* = 2 K (Fig. 3B and fig. S10, A and B). The same set of parameters reasonably reproduces the spectroscopic data at 10 K (Fig. 3B and fig. S10C). The rest of the parameters in Eqs. 4 and 6 are the same as those used in (*21*).

We fit the calculated resonance frequencies to experimental data by minimizing a cost function with the six free parameters. The cost function reads

$$C=\sum_{i}\frac{w_{i}}{N_{i}}\sum_{j\in i\text{th}\;\text{mode}}\frac{{\left(f_{\mathrm{ij}}-{\tilde{f}}_{\mathrm{ij}}\right)}^{2}}{{\tilde{f}}_{\mathrm{ij}}^{2}}$$ (22)

where *i* indicates the spectral modes that are fitted to; *N*_*i*_ is the number of experiment data points in the *i*th mode; *j* refers to the experiment data points in the *i*th mode; *f*_*ij*_ and ${\tilde{f}}_{\mathrm{ij}}$ are the calculated and measured resonance frequencies of the *i*th mode at *j*th data point, respectively; and *w*_*i*_ is the weight assigned to the *i*th mode. The fitting is performed over five different modes: four spectral modes [the quasi-ferromagnetic (qFM) magnon mode and qAFM modes of Fe^3+^ and two Er^3+^ modes] as a function of μ_0_*H* at 2 K as shown in fig. S10B, and one spectral mode (qAFM mode of Fe^3+^) as a function of *T* to obtain *T*_c_ at 0 T as shown in fig. S10A. The weights for the two Fe^3+^ (Er^3+^) modes versus μ_0_*H* are 0.3 (0.1), while it is 0.2 for the qAFM mode of Fe^3+^ versus *T*. The fit is not very sensitive to the choice of the weights. Figure S10C shows good agreement between experimental data and calculations at 10 K using the fitted parameter values, providing confidence to our fitting protocol.

### The extended Dicke model

Using spin-wave theory, the Fe^3+^ spins $S_{i}^{A/B}$ are rewritten in terms of a set of magnonic creation and annihilation operators (*a*_qFM_, *a*_qAFM_, $a_{\text{qFM}}^{†}$, and $a_{\text{qAFM}}^{†}$) (*21*, *34*), where the “qFM” (“qAFM”) corresponds to the *k* = 0 (*k* = π) quasi-momentum modes.

The magnon representation of Fe^3+^ spins is justified owing to the strong exchange interaction *J*_Fe_ = 4.96 meV, making it highly dispersive, while the Er^3+^ exchange interaction, *J*_Er_ = 0.0132 meV, is more than two orders of magnitude smaller. Hence, the Er^3+^ spins are well described by the collective spin approximation, and they couple only to the qAFM and qFM modes of Fe^3+^.

The derivation of the extended Dicke model from the spin Hamiltonian follows “Derivation of extended Dicke Hamiltonian” of (*21*). We briefly outline this here. The spin Hamiltonian is split in three parts, namely, ${\hat{H}}_{\text{Fe}}$, ${\hat{H}}_{\text{Er}}$, and ${\hat{H}}_{\text{Fe}-\text{Er}}$.

First, we rewrite the Fe^3+^ subsystem in terms of magnon annihilation and creation operators. The magnons are collective spin fluctuations above mean-field configuration for the bare Fe^3+^ subsystem. The ground state is the same as previous studies (*21*, *24*, *34*)

$${\bar{S}}_{0}^{A}=\left(\begin{array}{c} S\text{sin}\beta_{0}\\ 0\\ -S\text{cos}\beta_{0} \end{array}\right),\;{\bar{S}}_{0}^{B}=\left(\begin{array}{c} S\text{sin}\beta_{0}\\ 0\\ S\text{cos}\beta_{0} \end{array}\right)$$ (23)

where β_0_ is the mean-field canting angle of the Fe^3+^ spins from the *c* axis (*21*, *34*)

$$\beta_{0}=-\frac{1}{2}\text{arctan}\left[\frac{z_{\text{Fe}}D_{\text{Fe}}^{y}}{z_{\text{Fe}}J_{\text{Fe}}-A_{\text{Fe}}^{x}+A_{\text{Fe}}^{z}}\right]$$ (24)

We neglect the non-diagonal Fe^3+^-anisotropy term, $A_{\text{Fe}}^{\mathrm{xz}}$.

Spin-wave theory yields (*21*, *34*)

$${\hat{H}}_{\text{Fe}}\approx \sum_{k=0,\pi }\hbar \omega_{k}a_{k}^{†}a_{k}+\text{const}$$ (25)

with $\omega_{k}=g_{\text{Fe}}\mu_{B}/\hbar \sqrt{\left(b\text{cos}k-a\right)\left(d\text{cos}k+c\right)}$ and

$$a=\left[S/\left(g_{\text{Fe}}^{x}\mu_{B}\right)\right]\left[-A_{\text{Fe}}^{z}-A_{\text{Fe}}^{x}-\left(z_{\text{Fe}}J_{\text{Fe}}+A_{\text{Fe}}^{z}-A_{\text{Fe}}^{x}\right)\text{cos}\left(2\beta_{0}\right)+z_{\text{Fe}}D_{\text{Fe}}^{y}\text{sin}\left(2\beta_{0}\right)\right]$$ (26)

$$b=\left[S/\left(g_{\text{Fe}}^{x}\mu_{B}\right)\right]z_{\text{Fe}}J_{\text{Fe}}$$ (27)

$$c=\left[S/\left(g_{\text{Fe}}^{x}\mu_{B}\right)\right]\left[\left(z_{\text{Fe}}J_{\text{Fe}}+2A_{\text{Fe}}^{z}-2A_{\text{Fe}}^{x}\right)\text{cos}\left(2\beta_{0}\right)+z_{\text{Fe}}D_{\text{Fe}}^{y}\text{sin}\left(2\beta_{0}\right)\right]$$ (28)

$$d=\left[S/\left(g_{\text{Fe}}^{x}\mu_{B}\right)\right]\left[-z_{\text{Fe}}J_{\text{Fe}}\text{cos}\left(2\beta_{0}\right)-z_{\text{Fe}}D_{\text{Fe}}^{y}\text{sin}\left(2\beta_{0}\right)\right]$$ (29)

The derivation is as is from (*21*). First, we compute the Heisenberg equations of motion of S for $H_{\text{Fe}}$. Then, we linearize them by expressing $S_{i}^{A/B}={\bar{S}}_{0}^{A/B}+\delta S_{i}^{A/B}$. The effective Hamiltonian of the linearized equations of motion is decoupled harmonic oscillators. These harmonic oscillators are the magnons. The spin fluctuations in terms of ladder operators of the harmonic oscillators are

$$\delta S_{i}^{A}\approx \sqrt{\frac{S}{2N_{0}}}\left[\begin{array}{c} -\left(T_{0}-T_{\pi }\right)\text{cos}\beta_{0}\\ \left(Y_{0}-Y_{\pi }\right)\\ -\left(T_{0}-T_{\pi }\right)\text{sin}\beta_{0} \end{array}\right]$$ (30)

$$\delta S_{i}^{B}\approx \sqrt{\frac{S}{2N_{0}}}\left[\begin{array}{c} \left(T_{0}+T_{\pi }\right)\text{cos}\beta_{0}\\ \left(Y_{0}+Y_{\pi }\right)\\ -\left(T_{0}+T_{\pi }\right)\text{sin}\beta_{0} \end{array}\right]$$ (31)

where

$$T_{k}={\left(\frac{b\text{cos}k-a}{d\text{cos}k-a}\right)}^{1/4}\frac{a_{-k}^{†}+a_{k}}{\sqrt{2}}$$ (32)

$$Y_{k}={\left(\frac{d\text{cos}k+c}{b\text{cos}k-a}\right)}^{1/4}\frac{i\left(a_{-k}^{†}-a_{k}\right)}{\sqrt{2}}$$ (33)

We only keep *k* = 0 and π contributions, the qFM and qAFM modes of Fe^3+^ respectively, as there is negligible coupling to the other modes. Henceforth, these modes are labeled as *a*_qFM_ and *a*_qAFM_.

Next, we apply the collective spin approximation on the Er^3+^ spins

$$s_{i}^{s}=\frac{1}{N_{0}}\sum_{j=1}^{N_{0}}s_{j}^{s}$$ (34)

$$\equiv \frac{1}{N_{0}}\Sigma_{j}^{s}$$ (35)

finding

$$\begin{array}{c} {\hat{H}}_{\text{Er}}\approx \mu_{0}\mu_{B}g_{\text{Er}}^{x}H_{x}^{\text{DC}}\Sigma_{x}^{+}+z_{\text{Er}}J_{\text{Er}}\sum_{i=1}^{N_{0}}s_{i}^{A}\cdot \sum_{i\prime =1}^{N_{0}}\frac{s_{i^{\prime }}^{B}}{N_{0}}\\ -\sum_{i}\sum_{\xi =x,z}A_{\text{Er}}^{\xi }\sum_{s=A,B}{\left(\frac{1}{N_{0}}\sum_{j}s_{j,\xi }^{s}\right)}^{2} \end{array}$$ (36)

$$=\mu_{0}\mu_{B}g_{\text{Er}}^{x}H_{x}^{\text{DC}}\Sigma_{x}^{+}+\frac{z_{\text{Er}}J_{\text{Er}}}{N_{0}}\Sigma^{A}\cdot \Sigma^{B}-\sum_{s=A,B}\sum_{\xi =x,z}\frac{A_{\text{Er}}^{\xi }}{N_{0}}{\left(\Sigma_{\xi }^{s}\right)}^{2}$$ (37)

where $\Sigma_{\xi }^{\pm }=\Sigma_{\xi }^{A}\pm \Sigma_{\xi }^{B}$.

Last, we rewrite ${\hat{H}}_{\text{Fe}-\text{Er}}$ in terms of the spin-fluctuation operators of the Fe^3+^ spins and the collective spin operators of Er^3+^ spins to give

$$\begin{array}{cc} {\hat{H}}_{\text{Fe}-\text{Er}} & =4S\left(J\text{sin}\beta_{0}+D_{y}\text{cos}\beta_{0}\right)\Sigma_{x}^{+}+\left(-4{\mathrm{SD}}_{x}\text{cos}\beta_{0}\right)\Sigma_{y}^{-}\\ & +\sqrt{\frac{S}{N_{0}}}\left[(J\text{cos}\beta_{0}-D_{y}\text{sin}\beta_{0})T_{\pi }\Sigma_{x}^{+}+JY_{0}\Sigma_{y}^{+}\right.\\ & +\left.\left(D_{x}\text{sin}\beta_{0}\right)T_{\pi }\Sigma_{y}^{-}+D_{x}Y_{\pi }\Sigma_{z}^{-}-\left(J\text{sin}\beta_{0}+D_{y}\text{cos}\beta_{0}\right)T_{0}\Sigma_{z}^{+}\right] \end{array}$$ (38)

We have different prefactors of the parameters compared to (*21*) because they have extra factors of 1/2 as the Er^3+^ spins are modeled as 1/2σ^*s*^, whereas we absorb this in our spin variables $s^{s}$.

The complete spin-Dicke Hamiltonian, expressed in terms of the magnonic operators and collective Er^3+^ spin operators, is

$$\begin{array}{cc} {\hat{H}}_{\text{Dicke}}\approx & \sum_{m=\text{qFM},\text{qAFM}}\hbar \omega_{m}a_{m}^{†}a_{m}+E_{x}\Sigma_{x}^{+}+E_{y}\Sigma_{y}^{-}+\mu_{0}\mu_{B}g_{\text{Er}}^{x}H_{x}^{\text{DC}}\Sigma_{x}^{+}\\ & +\frac{z_{\text{Er}}J_{\text{Er}}}{N_{0}}\Sigma^{A}\cdot \Sigma^{B}-\sum_{\xi =x,z}\sum_{s=A,B}\frac{A_{\text{Er}}^{\xi }}{N_{0}}{\left(\Sigma_{\xi }^{s}\right)}^{2}+\frac{\hbar g_{x}}{\sqrt{N_{0}}}\left(a_{\text{qAFM}}^{†}+a_{\text{qAFM}}\right)\Sigma_{x}^{+}\\ & +\frac{i\hbar g_{y}}{\sqrt{N_{0}}}\left(a_{\text{qFM}}^{†}-a_{\text{qFM}}\right)\Sigma_{y}^{+}+\frac{\hbar g_{y\prime }}{\sqrt{N_{0}}}\left(a_{\text{qAFM}}^{†}+a_{\text{qAFM}}\right)\Sigma_{y}^{-}\\ & +\frac{i\hbar g_{z}}{\sqrt{N_{0}}}\left(a_{\text{qAFM}}^{†}-a_{\text{qAFM}}\right)\Sigma_{z}^{-}+\frac{\hbar g_{z\prime }}{\sqrt{N_{0}}}\left(a_{\text{qFM}}^{†}+a_{\text{qFM}}\right)\Sigma_{z}^{+} \end{array}$$ (39)

$E_{x}=4S\left(J\text{sin}\beta_{0}+D_{y}\text{cos}\beta_{0}\right)=h\times 0.0147341$ THz, $E_{y}=-4{\mathrm{SD}}_{x}\text{cos}\beta_{0}=h\times \left(-0.16999041\right)$ THz, and the five coupling strengths are

$$\hbar g_{x}=\sqrt{\mathrm{xS}}\left(J\text{cos}\beta_{0}-D_{y}\text{sin}\beta_{0}\right){\left(\frac{b+a}{d-c}\right)}^{1/4}=h\sqrt{x}\times 0.037\;\text{THz}$$ (40)

$$\hbar g_{y}=\sqrt{\mathrm{xS}}J{\left(\frac{d+c}{b-a}\right)}^{1/4}=h\sqrt{x}\times 0.031\;\text{THz}$$ (41)

$$\hbar g_{y\prime }=\sqrt{\mathrm{xS}}D_{x}\text{sin}\beta_{0}{\left(\frac{b+a}{d-c}\right)}^{1/4}=h\sqrt{x}\times 2.28\times 10^{-5}\;\text{THz}$$ (42)

$$\hbar g_{z}=\sqrt{\mathrm{xS}}D_{x}{\left(\frac{d-c}{b+a}\right)}^{1/4}=h\sqrt{x}\times 0.079\;\text{THz}$$ (43)

$$\hbar g_{z\prime }=\sqrt{\mathrm{xS}}\left(-J\text{sin}\beta_{0}-D_{y}\text{cos}\beta_{0}\right){\left(\frac{b-a}{d+c}\right)}^{1/4}=h\sqrt{x}\times -0.026109848\;\text{THz}$$ (44)

$x=\text{tanh}\left[\frac{|E_{x}+\mu_{0}\mu_{B}g_{\text{Er}}^{x}H_{x}^{\text{DC}}|}{2k_{B}T}\right]$ is the spin-dilution factor that incorporates the effect of temperature (*21*). We find that $\langle \Sigma_{y}^{\pm }\rangle \approx 0$. The role of the bare bosonic frequency, $\omega_{0}$, is played by $\omega_{\text{qAFM}}=2\pi \times 0.956$ THz and that of the bare atomic frequency, $\omega_{a}$, is played by $\mu_{B}\mu_{0}g_{\text{Er}}^{x}H_{x}^{\text{DC}}/\hbar$ and, hence, controlled by the magnetic field. The largest coupling constant, $g_{z}$, dictates the important Dicke physics of the SRPT. The other coupling constants merely fine-tune the exact location of the SRPT. We note that the magnon mode responsible for the SRPT is not the qFM mode but the qAFM mode. The qFM mode couples with the $\Sigma_{z}^{+}$ component with a coupling strength of $g_{z\prime }$ that is small compared with other coupling strengths. Additionally, because our experiments have been performed in ${\vec{H}}_{\text{DC}}∥a$, the $\langle \Sigma_{z}^{+}\rangle$ is negligible in the ground state in both Γ_12_ and Γ_2_ phases. Therefore, the contribution of qFM mode is negligible or at most would contribute to the renormalization of the SR-phase boundary.

## Supplementary Materials

### This PDF file includes:

Supplementary Text Figs. S1 to S11 Table S1 References
