# Supplementary material for: Observation of the magnonic Dicke superradiant phase transition
Source: Sci Adv. 2025 Apr 4;11(14):eadt1691. doi: 10.1126/sciadv.adt1691 (PMC13155478; doi:10.1126/sciadv.adt1691)
Supplement: Supplementary file 1 — Supplementary Text Figs. S1 to S11 Table S1 References [file sciadv.adt1691_sm.pdf]

Science Advances
AAAS

Supplementary Materials for

Observation of the magnonic Dicke superradiant phase transition

Dasom Kim *et al.*

Corresponding author: Shixun Cao, sxcao@shu.edu.cn; Junichiro Kono, kono@rice.edu

Sci. Adv. **11**, eadt1691 (2025)
DOI: 10.1126/sciadv.adt1691

This PDF file includes:

Supplementary Text
Figs. S1 to S11
Table S1
References

Supplementary Text

S1. SRPT at finite detuning

An anti-crossing of two polaritons occurs at the zero-detuning point ($\omega_0 = \omega_a$). When the normalized coupling strength ($\eta \equiv g/\omega_0$) reaches the critical value of 0.5, the system undergoes the SRPT, and ω_- becomes zero. As shown in fig. S1A, for $\eta = 0.5$ the SRPT occurs when $\omega_0 = \omega_a$. For $\eta = 0.1$ (fig. S1B), a situation more comparable to $ErFeO_3$, we find the phase boundary moves to the $\omega_a < \omega_0$, while the anti-crossing still occurs at the zero-detuning point. Thus one can achieve the SRPTs with a small η as long as $\nu \equiv \omega_a/\omega_0$ is small enough to satisfy the Eq. 2. By contrast, when $\nu > 1$, the η_c becomes higher than 0.5.

S2. THz absorption spectra at high temperatures

Figure S6A shows temperature-dependent absorption spectra of the qAFM mode of Fe^{3+}. The kink occurs at 4 K which is the SR-phase boundary at 0 T. Below this temperature, the Fe^{3+} order parameter $\langle S_y^{A/B} \rangle$ becomes finite. Figure S6B shows magnetic field dependence of the qAFM mode of Fe^{3+} at 10 K. Only a slight change was observed at low magnetic fields without any signature of the phase transition, consistent with our phase diagram. Meanwhile, two modes that emerge at high fields are Er^{3+} EPR modes. As described in main text, $ErFeO_3$ can be modeled by the two-sublattice model. This implies we should expect four modes in total: the qFM and qAFM modes for Fe^{3+} spins, and in-phase and out-of-phase EPR modes for Er^{3+} spins. Here, we are considering the relative phase of precession of two Er^{3+} spins. A detailed derivation is in Materials and Methods and follows that in Ref. (*21*). Our theory finds the lowest mode is the out-of-phase mode that is coupled to qAFM, establishing a magnon-spin system. Due to the polarization selection rule described in Fig. 2B, the qFM mode does not appear in fig. S6.

S3. Spectroscopic evidence of hybridization at zero-detuning

A direct spectroscopic signature of the hybridization of the Fe^{3+} qAFM mode and the lower Er^{3+} EPR mode would be an anticrossing behavior in the frequencies, $\omega_\pm$, of the upper- and lower-polariton branches, as a function of magnetic field, H_{DC}. Figure S2 shows calculated $\omega_\pm$ in H_{DC} up to 20 T, exhibiting an anticrossing behavior around the zero-detuning point at ∼17 T. However,

experimentally, the field range of the THz time-domain magnetospectroscopy setup used was limited to 7 T in the Voigt geometry ($\vec{k}_{\rm THz} \perp \vec{H}_{\rm DC}, \vec{k}_{\rm THz} \parallel b$), which is required for realizing a $\vec{H}_{\rm THz} \parallel \vec{H}_{\rm DC} \parallel a$ configuration needed for a b-cut crystal. Nonetheless, we conducted experiments in the Faraday geometry ($\vec{k}_{\rm THz} \parallel \vec{H}_{\rm DC} \parallel c$, $\vec{H}_{\rm THz} \parallel a$) with a c-cut crystal and were able to observe an anticrossing. This was possible because the z component of the Landé g-factor of Er^{3+} spins is larger than the x component, which lowers the zero-detuning magnetic field to an accessible range. Figure S3 shows transmission decrease as a function of magnetic field and frequency at 1.4 K, in which a pronounced anticrossing is observed at around 7 T. This coupling is modeled by g_z (or D_x) in Eqs. 11, 39, and 43, which is a coupling between the qAFM and the Er^{3+} EPR mode (out-of-phase). This is the coupling that is responsible for the $\Gamma_2 \rightarrow \Gamma_{12}$ phase transition, highlighting the importance of hybridization in the phase transition. We also note that the brightest mode (the solid line) is the in-phase Er^{3+} EPR mode.

S4. Justification of the magnonic SRPT

We provide additional justification as to why the phase transition we observed, described by the extended Dicek model, is indeed equivalent to the conventional Dicke SRPT. As stated earlier, the most important term for Dicke-type interaction is the x-component of the Fe^{3+}–Er^{3+} antisymmetric exchange interaction, D_x. This becomes clear if we set all the other Fe^{3+}–Er^{3+} couplings (J and D_y) and all the Er^{3+}-spin terms ($J_{\rm Er}$ and $A_{\rm Er}^{x/z}$) to be zero; then the extended Dicke model of Eq. 39 becomes the simple Dicke model. We demonstrate our claim that the observed phases and SRPT are equivalent to the simple Dicke model by showing that the phase diagram of the extended Dicke model is adiabatically connected to that of the simple Dicke model.

Figure S4 shows this equivalence. We define a parameter λ that interpolates between the extended Dicke Hamiltonian ($\lambda = 1$) and the simple Dicke model ($\lambda = 0$). Specifically, we define a Hamiltonian extending Eq. 39 such that

$$H_{\rm interp}[\lambda] = H[J_{\rm Fe}, D_{\rm Fe}^{y}, A_{\rm Fe}^{x/z}, D_x, \lambda J_{\rm Er}, \lambda A_{\rm Er}^{x/z}, \lambda J, \lambda D_y]. \tag{S1}$$

We plot the Fe^{3+}-canting order parameter, $\langle S_y^{\rm A/B} \rangle$ at $H_{\rm DC} = 0$ as functions of D_x and λ in fig. S4A. In figs. S4A and B, we see that the ordered and disordered phases of the extended Dicke model ($\lambda = 1$) continuously evolve into those of the simple Dicke model ($\lambda = 0$). The top panel of fig. S4C

shows the $\lambda = 0$ cut of fig. S4A where two order parameters (Fe^{3+} and Er^{3+}) are finite above a certain critical point, and highlights the important role of D_x. The $D_x = 0$ cut reveals that while the Er^{3+} order parameter is finite above a certain critical point, the Fe^{3+} order parameter is always zero in the absence of the D_x interaction, highlighting the necessity of D_x (fig. S4C, bottom panel). Figure S4A shows a trajectory smoothly connects a conventional Dicke SR phase (green star) to the experimental SR phase (blue star). Figure S4B shows a critical magnetic field strength along the way from the green star to the blue star, which confirms the adiabatic connection of our SR phase to the conventional SR phase.

The "non-Dicke" terms do not change the qualitative features of the spectrum. To show how the additional terms in the extended Dicke model affect the phase transition, we set a finite value of $D_x = 0.045$ meV, and plot the spectrum for $\lambda = 0$ (fig. S5). With the D_x term alone, we confirm the occurence of the SRPT, and the kink and softening in the two polariton branches, respectively. Subsequently, we set each additional terms to a non-zero value one at a time, and observe how the critical magnetic field shifts. Figure S5 shows reduced the size of the SR phase with the inclusion of the D_y, A^x_{Er}, and J terms. It is the J term that governs the $\sim \mathbf{S} \cdot \mathfrak{s}$ interaction. It also shows an increased size of the SR phase with the inclusion of the J_{Er} term (atom–atom interaction) and A^z_{Er} term. We conclude that the J_{Er} and A^z_{Er} terms enhance the SR phase, whereas the J, D_y, and A^x_{Er} terms suppress it.

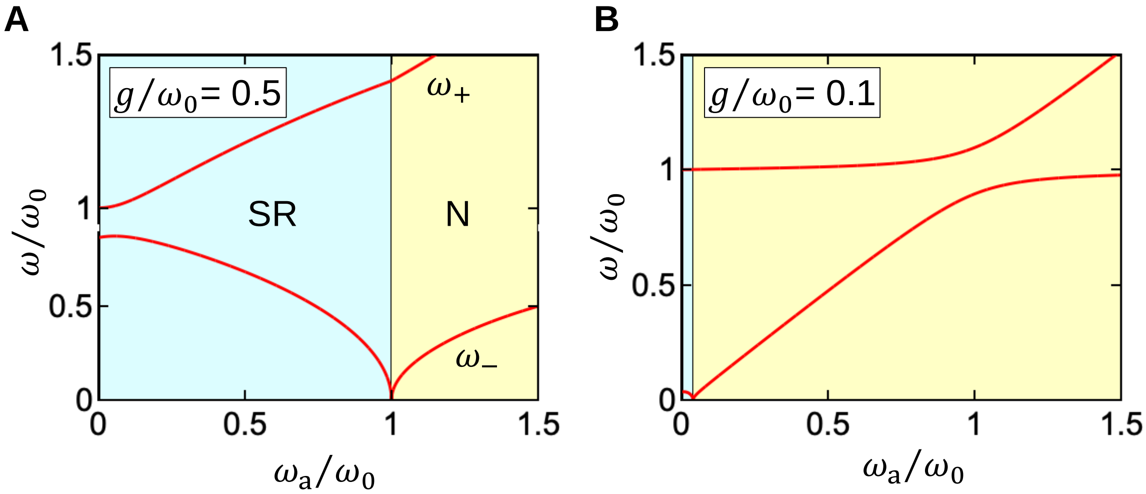

Figure S1: **Occurrence of the superradiant phase transition at finite detuning.** (**A** and **B**), Normalized frequencies of the upper-polariton (ω_+) and lower-polariton (ω_-) modes as a function of ω_a/ω_0 calculated using the Dicke model without the A^2 term with $g/\omega_0 = 0.5$ (**A**) and with $g/\omega_0 = 0.1$ (**B**).

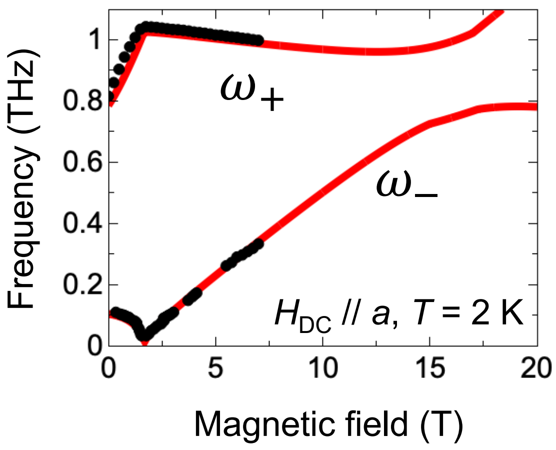

Figure S2: **Calculated resonance frequencies of the upper- and lower-polariton branches, ω_+ and ω_-, respectively, as a function of magnetic field up to 20 T applied along the a-axis.** An anticrossing behavior is seen around the zero-detuning point at ~17 T. Solid circles: experimental data.

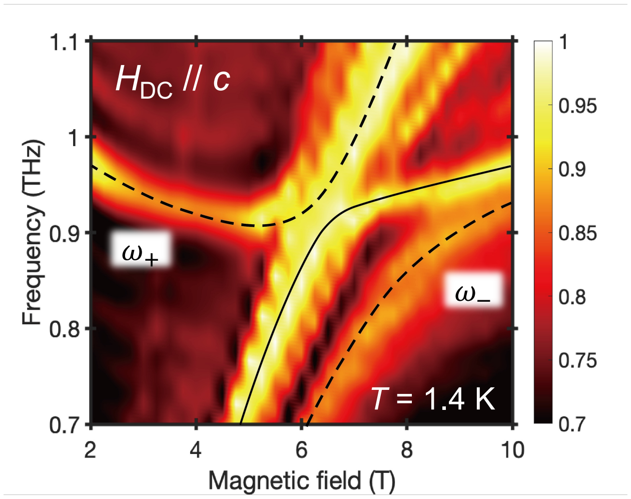

Figure S3: **Transmission decrease** $(1-\tilde{T})$ **as a function of magnetic field applied parallel to the** c **axis at 1.4 K.** The Fe^{3+} qAFM and Er^{3+} EPR modes are hybridized, creating the upper- and lower-polariton branches ($\omega_\pm$); the lines are a guide to the eye.

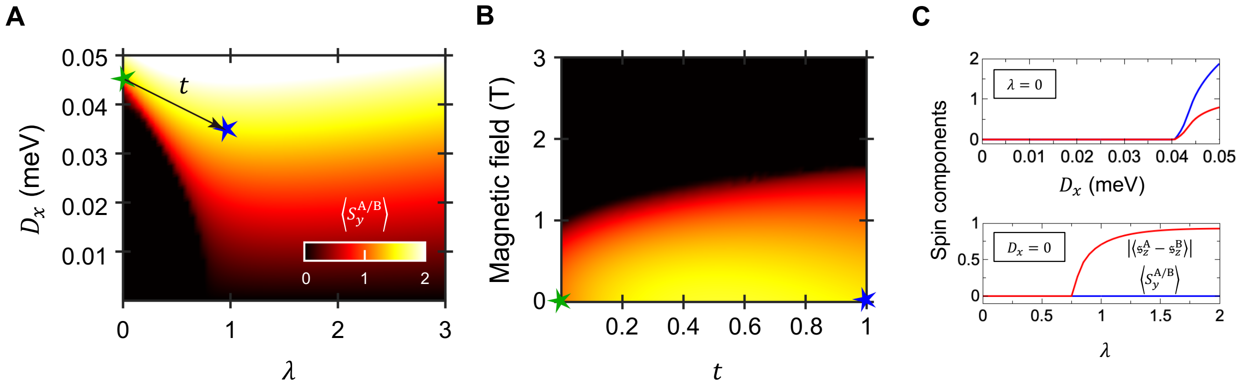

Figure S4: $T = 2$ **K zero-field phase diagram.** (**A**) The Fe^{3+} order parameters as a function of D_x and λ. (**B**) The Fe^{3+} order parameters as a function of external magnetic field and the parametric variable t, which parameterizes the λ-D_x trajectory shown in (**A**). (**C**) (Top) $\lambda = 0$ cut of (**A**), (bottom) $D_x = 0$ cut of (**A**). We can see clearly that the Fe^{3+} order parameters are independent of varying of λ.

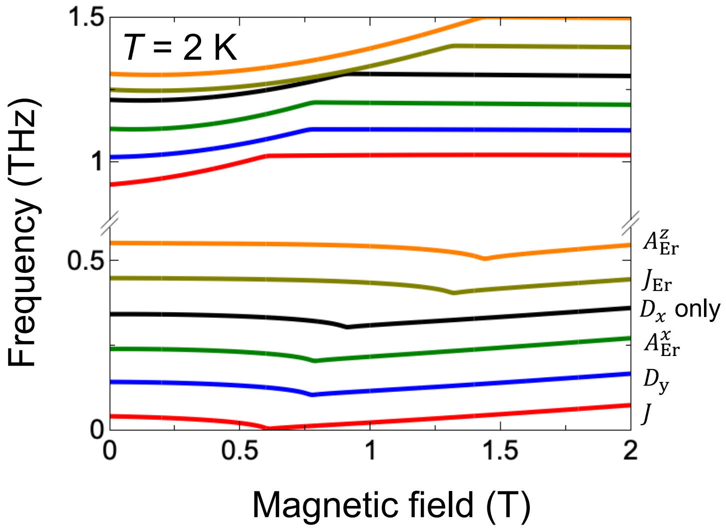

Figure S5: **Effects of the "non-Dicke" parameters on the polariton spectra calculated in $\vec{H}_{DC} \parallel a$ at 2 K.** The upper six curves are upper-polariton branches and the lower six curves are corresponding lower-polariton branches. The curves are offset by 0.1 for clarity. J_{Fe}, D_{Fe}^x, and $A_{Fe}^{x/z}$ are the same as provided in the main text. D_x only: $D_x = 0.045$ meV and $J = D_y = J_{Er} = A_{Er}^x = A_{Er}^z = 0$; J, D_y, and J_{Er}: Addition of J, D_y, and J_{Er} (twice smaller than the value shown in table S1) to the D_x only case, respectively; $A_{Er}^{x/z}$: Addition of $A_{Er}^{x/z}$ (five times smaller than the value shown in table S1) to the D_x only case. The addition of J, D_y, and A_{Er}^x weakens the SR-phase boundary. The addition of J_{Er} and A_{Er}^z strengthens the SR-phase boundary.

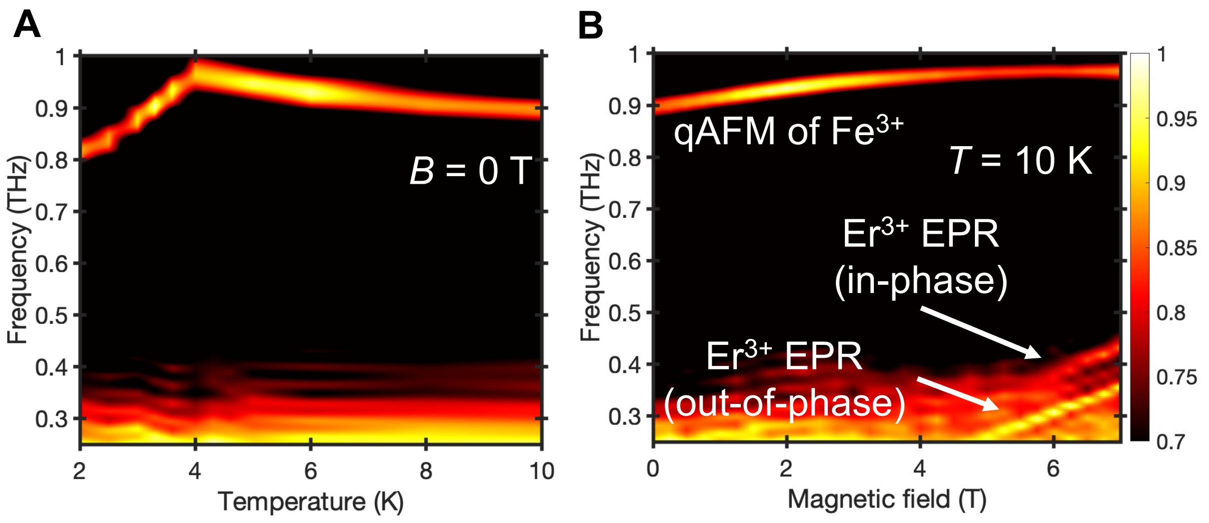

Figure S6: **Terahertz transmission decrease** $(1-\tilde{T})$ **spectra.** (**A**) transmission decrease as a function of temperature in THz-TDS, showing a kink at the phase boundary. (**B**) transmission decrease as a function of the magnetic field in THz-TDMS. The qAFM mode of Fe^{3+} and two Er^{3+} EPR modes are observed. Fig. 3B (right panel) plots the out-of-phase mode. Our mean-field calculation in Fig. S10C shows all three modes.

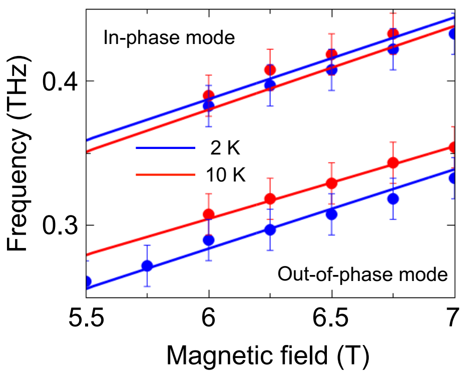

Figure S7: **In-phase and out-of-phase modes of Er^{3+} spins below and above the critical temperature, showing shifts only for the 2 K case.** Blue color: 2 K, red color: 10 K, lines: theory, circles: experimental data. The error bars indicate a frequency resolution determined by the time window ($\Delta f = 1/33$ ps $= 0.0286$ THz).

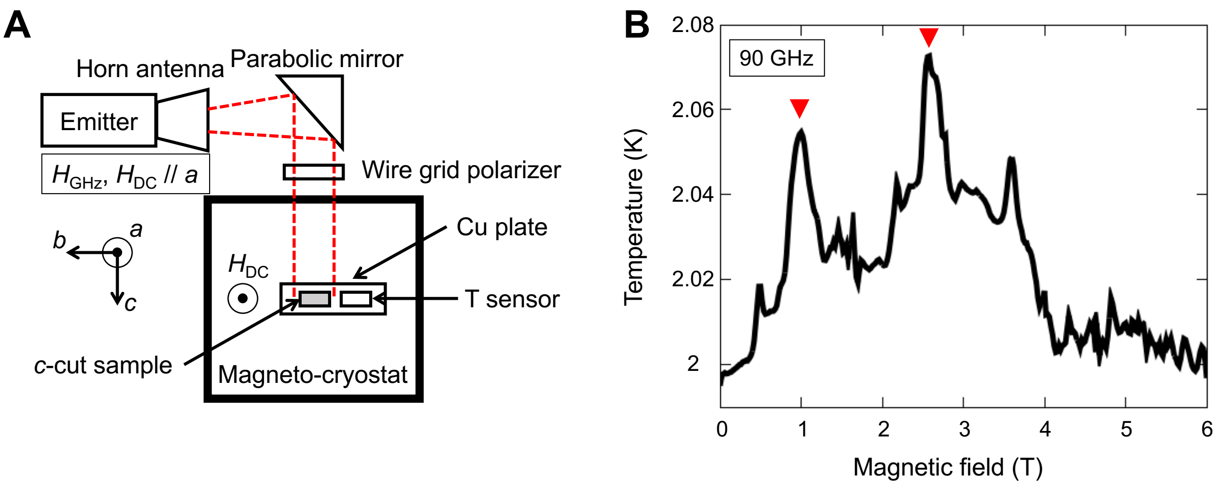

Figure S8: **Thermal detection of Er^{3+} EPR modes.** (**A**) A schematic of the setup for thermal detection. (**B**) The sample temperature as a function of the static magnetic field with 90 GHz illumination. The temperature increases when the incident photon energy coincides with the transition energy of magnetic resonances.

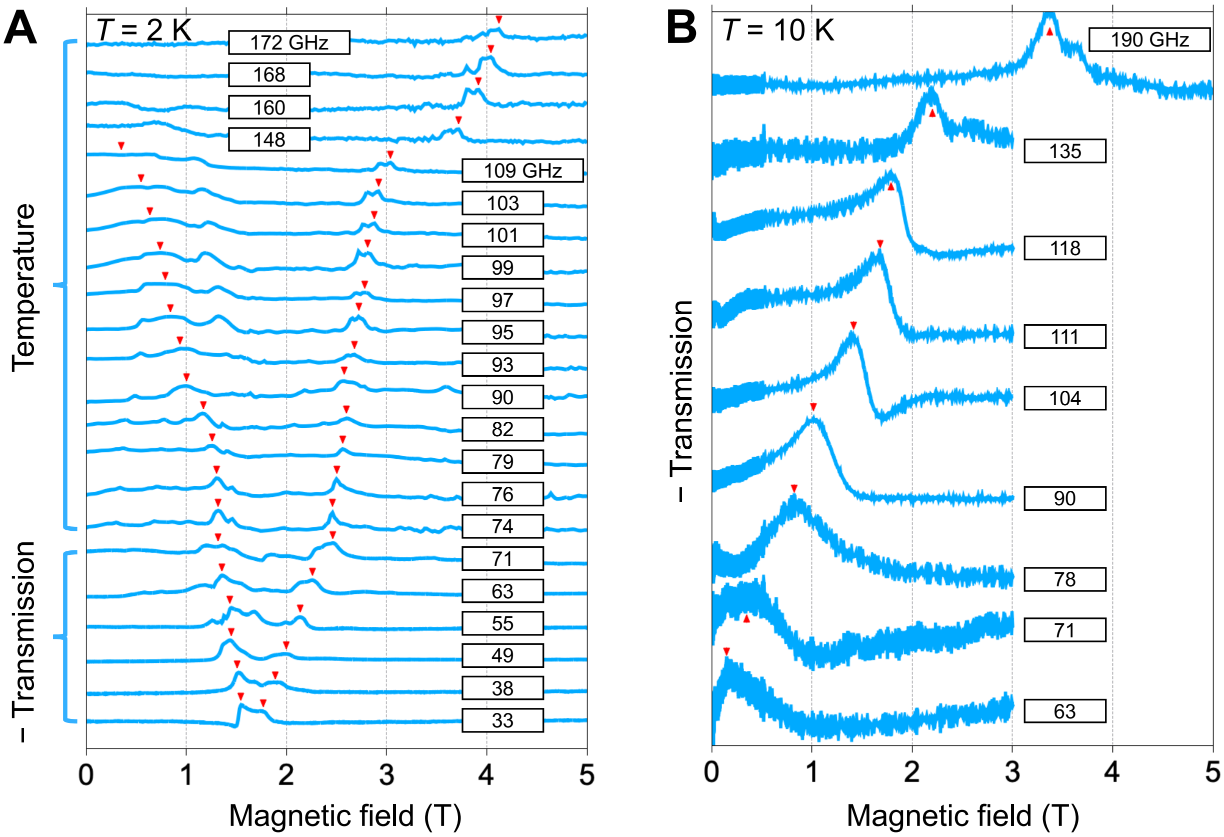

Figure S9: **Raw data of GHz measurements with 0-to-1 scale.** (**A**) From 33 to 71 GHz (74 to 172 GHz), transmission (temperature) spectra as a function of the magnetic field at 2 K. These data are used to generate the two middle and bottom panels in Fig. 2C. (**B**) From 63 to 190 GHz, transmission spectra as a function of the magnetic field at 10 K. Red triangles indicate the resonance peak positions in Fig. 3B (right panel, red circles).

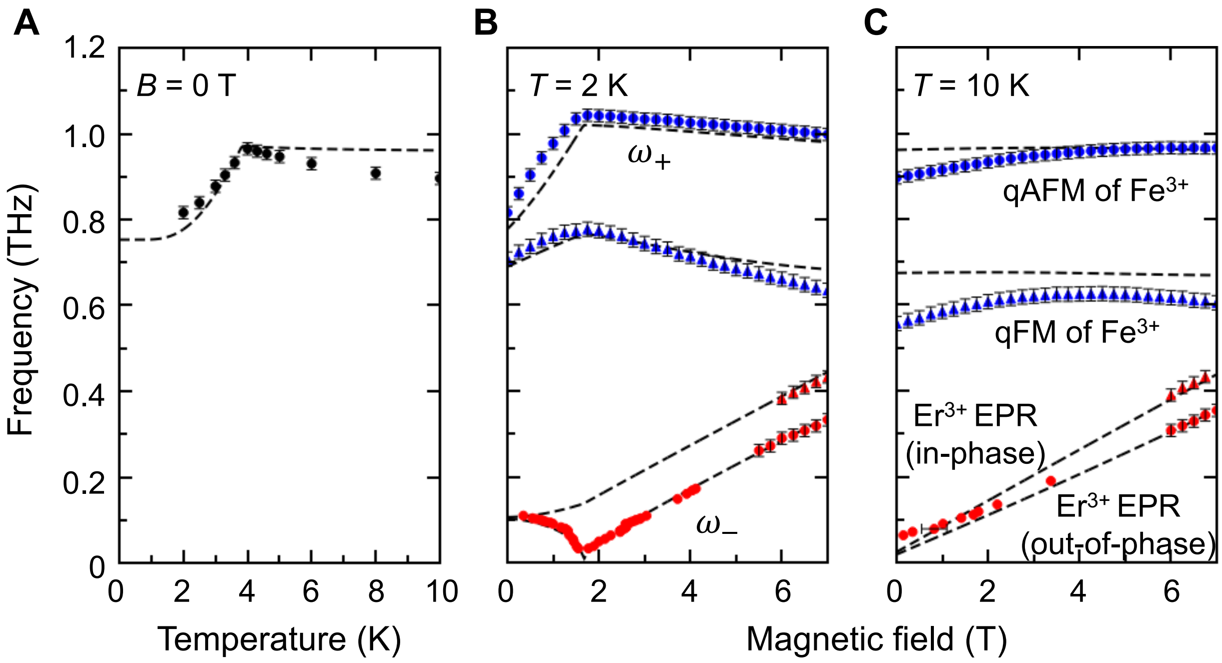

Figure S10: **Comparison between experimental data and fitting results.** (**A**) Temperature dependent absorption peaks of the qAFM mode of Fe^{3+} extracted from Fig. S6A. (**B** and **C**) Magnetic field dependent absorption peaks of all four modes at 2 K (**B**) and at 10 K (**C**). We present our fitting curves in (**A**) and (**B**), and calculations for 10 K in **(C)**. The blue circles, red triangles, and red circles in (**B**) are extracted from Fig. 2C, while those in (**C**) are from fig. S6B. The blue triangles correspond to the qFM mode of Fe^{3+}, obtained from separate experiments with a 90° rotated incident THz magnetic field polarization. The vertical error bars indicate a frequency resolution determined by the time window ($\Delta f = 1/30$ ps = 0.0333 THz for the qFM mode and $\Delta f = 1/35$ ps = 0.0286 THz for all other modes). The horizontal error bar indicates a standard deviation.

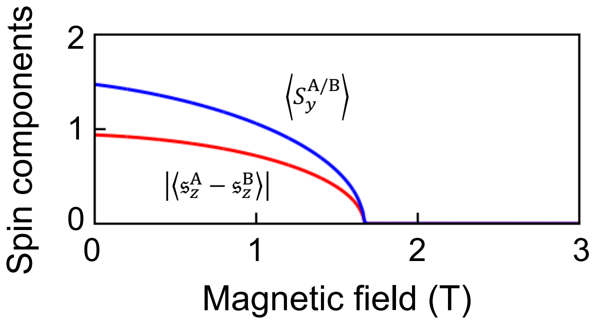

Figure S11: **Two order parameters evidencing the magnonic SRPT calculated at 2 K in the presence of the magnetic field.**

Table S1: **Mean-field model parameters from fitting and Ref. (*21*).** Listed parameter uncertainties are errors of fit, determined by the method in Ref. (*55*).

Fe^{3+} subsystem	Er^{3+} subsystem	Fe^{3+}–Er^{3+} interaction
$J_{Fe} = 4.96$ meV	$J_{Er} = 0.01328(5)$ meV	$J = 0.6$ meV
$D_{Fe}^{y} = -0.107$ meV	$A_{Er}^{x} = 0.124(4)$ meV	$D_x = 0.034$ meV
$A_{Fe}^{x} = 0.0073$ meV	$A_{Er}^{z} = 0.1480(3)$ meV	$D_y = 0.003$ meV
$A_{Fe}^{z} = 0.0176(3)$ meV	$A_{Er}^{xz} = 0$ meV	
$A_{Fe}^{xz} = 0$ meV	$\mathfrak{g}_{Er}^{x} = 4.16(8)$	
$\mathfrak{g}_{Fe}^{x} = 3.5734(3)$	$\mathfrak{g}_{Er}^{y} = 3.4$	
$\mathfrak{g}_{Fe}^{y} = 2$	$\mathfrak{g}_{Er}^{z} = 9.6$	
$\mathfrak{g}_{Fe}^{z} = 0.6$		

REFERENCES AND NOTES

1. R. H. Dicke, Coherence in spontaneous radiation processes. *Phys. Rev.* **93**, 99–110 (1954).

2. B. M. Garraway, The Dicke model in quantum optics: Dicke model revisited. *Philos. Transact. A Math. Phys. Eng. Sci.*. **369**, 1137–1155 (2011).

3. P. Forn-Díaz, L. Lamata, E. Rico, J. Kono, E. Solano, Ultrastrong coupling regimes of light-matter interaction. *Rev. Mod. Phys.* **91**, 025005 (2019).

4. A. Frisk Kockum, A. Miranowicz, S. De Liberato, S. Savasta, F. Nori, Ultrastrong coupling between light and matter. *Nat. Rev. Phys.* **1**, 19–40 (2019).

5. K. Cong, Q. Zhang, Y. Wang, G. T. Noe, A. Belyanin, J. Kono, Dicke superradiance in solids. *J. Opt. Soc. Am. B* **33**, C80–C101 (2016).

6. N. Marquez Peraca, A. Baydin, W. Gao, M. Bamba, J. Kono, "Ultrastrong light-matter coupling in semiconductors" in *Semiconductor Quantum Science and Technology*, M. Kira, S. T. Cundiff, Eds. (Elsevier, 2020), vol. **105**, pp. 89–151.

7. F. Schlawin, D. M. Kennes, M. A. Sentef, Cavity quantum materials. *Appl. Phys. Rev.* **9**, 011312 (2022).

8. V. Buzžek, M. Orszag, M. Rosško, Instability and entanglement of the ground state of the Dicke model. *Phys. Rev. Lett.* **94**, 163601 (2005).

9. K. Hepp, E. H. Lieb, On the superradiant phase transition for molecules in a quantized radiation field: The Dicke maser model. *Ann. Phys.* **76**, 360–404 (1973).

10. Y. K. Wang, F. T. Hioe, Phase transition in the Dicke model of superradiance. *Phys. Rev. A* **7**, 831–836 (1973).

11. D. S. Shapiro, W. V. Pogosov, Y. E. Lozovik, Universal fluctuations and squeezing in a generalized Dicke model near the superradiant phase transition. *Phys. Rev. A* **102**, 023703 (2020).

12. P. Nataf, C. Ciuti, No-go theorem for superradiant quantum phase transitions in cavity QED and counter-example in Circuit QED. *Nat. Commun.* **1**, 72 (2010).

13. M. Bamba, K. Inomata, Y. Nakamura, Superradiant phase transition in a superconducting circuit in thermal equilibrium. *Phys. Rev. Lett.* **117**, 173601 (2016).

14. P. Kirton, M. M. Roses, J. Keeling, E. G. Dalla Torre, Introduction to the Dicke model: From equilibrium to nonequilibrium, and *vice versa*. *Adv. Quantum Technol.* **2**, 1800043 (2019).

15. L. Garziano, A. Settineri, O. Di Stefano, S. Savasta, F. Nori, Gauge invariance of the Dicke and Hopfield models. *Phys. Rev. A* **102**, 023718 (2020).

16. D. Lamberto, O. D. Stefano, S. Hughes, F. Nori, S. Savasta, Quantum phase transitions in many-dipole light-matter systems. arXiv:2405.10711 (2024).

17. D. Lamberto, G. Orlando, S. Savasta, Superradiant quantum phase transition in open systems: System-bath interaction at the critical point. arXiv:2411.16514 (2024).

18. K. Rzażewski, K. Wódkiewicz, W. Żakowicz, Phase transitions, two-level atoms, and the A^2 term. *Phys. Rev. Lett.* **35**, 432–434 (1975).

19. G. M. Andolina, F. M. D. Pellegrino, V. Giovannetti, A. H. MacDonald, M. Polini, Cavity quantum electrodynamics of strongly correlated electron systems: A no-go theorem for photon condensation. *Phys. Rev. B* **100**, 121109 (2019).

20. P. Nataf, T. Champel, G. Blatter, D. M. Basko, Rashba cavity QED: A route towards the superradiant quantum phase transition. *Phys. Rev. Lett.* **123**, 207402 (2019).

21. M. Bamba, X. Li, N. Marquez Peraca, J. Kono, Magnonic superradiant phase transition. *Commun. Phys.* **5**, 3 (2022).

22. X. Li, M. Bamba, Q. Zhang, S. Fallahi, G. C. Gardner, W. Gao, M. Lou, K. Yoshioka, M. J. Manfra, J. Kono, Vacuum Bloch–Siegert shift in Landau polaritons with ultra-high cooperativity. *Nat. Photonics* **12**, 324–329 (2018).

23. S. Hughes, C. Gustin, F. Nori, Reconciling quantum and classical spectral theories of ultrastrong coupling: Role of cavity bath coupling and gauge corrections. *Opt. Quantum* **2**, 133–139 (2024).

24. X. Li, M. Bamba, N. Yuan, Q. Zhang, Y. Zhao, M. Xiang, K. Xu, Z. Jin, W. Ren, G. Ma, S. Cao, D. Turchinovich, J. Kono, Observation of Dicke cooperativity in magnetic interactions. *Science* **361**, 794–797 (2018).

25. N. Marquez Peraca, X. Li, J. M. Moya, K. Hayashida, D. Kim, X. Ma, K. J. Neubauer, D. F. Padilla, C. L. Huang, P. Dai, A. H. Nevidomskyy, H. Pu, E. Morosan, S. Cao, M. Bamba, J. Kono, Quantum simulation of an extended Dicke model with a magnetic solid. *Commun. Mater.* **5**, 42 (2024).

26. T. Makihara, K. Hayashida, G. T. Noe II, X. Li, N. Marquez Peraca, X. Ma, Z. Jin, W. Ren, G. Ma, I. Katayama, J. Takeda, H. Nojiri, D. Turchinovich, S. Cao, M. Bamba, J. Kono, Ultrastrong magnon–magnon coupling dominated by antiresonant interactions. *Nat. Commun.* **12**, 3115 (2021).

27. K. Hayashida, T. Makihara, N. Marquez Peraca, D. Fallas Padilla, H. Pu, J. Kono, M. Bamba, Perfect intrinsic squeezing at the superradiant phase transition critical point. *Sci. Rep.* **13**, 2526 (2023).

28. J. M. Lee, H.-W. Lee, M.-J. Hwang, Cavity magnonics with easy-axis ferromagnets: Critically enhanced magnon squeezing and light-matter interaction. *Phys. Rev. B* **108**, L241404 (2023).

29. H. Yuan, Y. Cao, A. Kamra, R. A. Duine, P. Yan, Quantum magnonics: When magnon spintronics meets quantum information science. *Phys. Rep.* **965**, 1–74 (2022).

30. L. Liensberger, A. Kamra, H. Maier-Flaig, S. Geprägs, A. Erb, S. T. B. Goennenwein, R. Gross, W. Belzig, H. Huebl, M. Weiler, Exchange-enhanced ultrastrong magnon-magnon coupling in a compensated ferrimagnet. *Phys. Rev. Lett.* **123**, 117204 (2019).

31. A. Kamra, W. Belzig, A. Brataas, Magnon-squeezing as a niche of quantum magnonics. *Appl. Phys. Lett.* **117**, 090501 (2020).

32. C. Emary, T. Brandes, Quantum chaos triggered by precursors of a quantum phase transition: The Dicke model. *Phys. Rev. Lett.* **90**, 044101 (2003).

33. X. Li, D. Kim, Y. Liu, J. Kono, Terahertz spin dynamics in rare-earth orthoferrites. *Photon. Insights* **1**, R05 (2022).

34. G. Herrmann, Resonance and high frequency susceptibility in canted antiferromagnetic substances. *J. Phys. Chem. Solid* **24**, 597–606 (1963).

35. V. Klochan, N. Kovtun, V. Khmara, Low-temperature spin configuration of iron ions in erbium orthoferrite. *Sov. Phys. JETP* **41**, 357 (1975).

36. M. P. Zic, W. T. Fuhrman, K. Wang, S. Ran, J. Paglione, N. P. Butch, Coupled spin waves and crystalline electric field levels in candidate multiferroic $ErFeO_3$. *J. Appl. Phys.* **130**, 014102 (2021).

37. R. L. White, Review of recent work on the magnetic and spectroscopic properties of the rare-earth orthoferrites. *J. Appl. Phys.* **40**, 1061–1069 (1969).

38. A. M. Kadomtseva, I. B. Krynetskiĭ, V. M. Matveev, Nature of the spontaneous and field-induced low-temperature orientational transitions in erbium orthoferrite. *J. Exp. Theor. Phys.* **52**, 732–737 (1980).

39. I. M. Vitebskiĭ, N. K. Danshin, A. I. Izotov, M. A. Sdvizhkov, L. T. Tsymbal, Anomalous critical dynamics of a low-temperature transition in erbium orthoferrite. *J. Exp. Theor. Phys.* **71**, 187–190 (1990).

40. A. Baydin, T. Makihara, N. M. Peraca, J. Kono, Time-domain terahertz spectroscopy in high magnetic fields. *Front. Optoelectron.* **14**, 110–129 (2021).

41. G. F. Herrmann, Magnetic resonances and susceptibility in orthoferrites. *Phys. Rev.* **133**, A1334 (1964).

42. K. Yamaguchi, T. Kurihara, Y. Minami, M. Nakajima, T. Suemoto, Terahertz time-domain observation of spin reorientation in orthoferrite $ErFeO_3$ through magnetic free induction decay. *Phys. Rev. Lett.* **110**, 137204 (2013).

43. V. G. Baryakhtar, I. M. Vitebeskiĭ, D. A. Yablonskiĭ, Theory of metamagnetic phase transitions. *Sov. Phys. Solid State* **19**, 1249–1254 (1977).

44. N. K. Danshin, V. N. Derkachenko, N. M. Kovtun, M. A. Sdvizhkov, Metamagnetic phase transition in $ErFeO_3$. *Sov. Phys. Solid State* **28**, 1461–1464 (1986).

45. D. L. Wood, L. M. Holmes, J. P. Remeika, Exchange fields and optical Zeeman effect in $ErFeO_3$. *Phys. Rev.* **185**, 689–695 (1969).

46. A. K. Zvezdin, A. A. Muchin, A. I. Popov, Magnetic-structure instability resulting from the intersection of energy levels. *JETP Lett.* **23**, 240 (1976).

47. D. Porras, P. A. Ivanov, F. Schmidt-Kaler, Quantum simulation of the cooperative Jahn-Teller transition in 1D ion crystals. *Phys. Rev. Lett.* **108**, 235701 (2012).

48. M. Hagiwara, K. Katsumata, H. Yamaguchi, M. Tokunaga, I. Yamada, M. Gross, P. Goy, A complete frequency-field chart for the antiferromagnetic resonance in MnF_2. *Int. J. Infrared and Mill.* **20**, 617–622 (1999).

49. X. X. Zhang, Z. C. Xia, Y. J. Ke, X. Q. Zhang, Z. H. Cheng, Z. W. Ouyang, J. F. Wang, S. Huang, F. Yang, Y. J. Song, G. L. Xiao, H. Deng, D. Q. Jiang, Magnetic behavior and complete high-field magnetic phase diagram of the orthoferrite $ErFeO_3$. *Phys. Rev. B* **100**, 054418 (2019).

50. K. P. Belov, A. K. Zvezdin, A. M. Kadomtseva, R. Z. Levitin, Spin-reorientation transitions in rare-earth magnets. *Sov. Phys. Usp.* **19**, 574–596 (1976).

51. A. M. Balbashov, G. V. Kozlov, A. A. Mukhin, A. S. Prokhorov, "Submillimeter spectroscopy of antiferromagnetic dielectrics: Rare-earth orthoferrites" in *High Frequency Processes in Magnetic Materials* (World Scientific Publishing, 1995), pp. 56–91.

52. V. D. Buchelnikov, N. K. Danshin, L. T. Tsymbal, V. G. Shavrov, Magnetoacoustics of rare-earth orthoferrites. *Phys Uspekhi* **39**, 547–572 (1996).

53. J. Zhang, "Microwave spectroscopy on two dimensional electron/hole gases," thesis, Rice University, Houston, TX (2018).

54. T. E. Kritzell, A. Baydin, F. Tay, R. Rodriguez, J. Doumani, H. Nojiri, H. O. Everitt, I. Barsukov, J. Kono, Terahertz cavity magnon polaritons. *Adv. Opt. Mater.* **12**, 2302270 (2024).

55. K. W. Vugrin, L. P. Swiler, R. M. Roberts, N. J. Stucky-Mack, S. P. Sullivan, Confidence region estimation techniques for nonlinear regression in groundwater flow: Three case studies. *Water Resour. Res.* **43**, W03423 (2007).
